# Frequent first-trimester pregnancy loss in rhesus macaques infected with African-lineage Zika virus

**DOI:** 10.1371/journal.ppat.1011282

**Published:** 2023-03-28

**Authors:** Jenna R. Rosinski, Lauren E. Raasch, Patrick Barros Tiburcio, Meghan E. Breitbach, Phoenix M. Shepherd, Keisuke Yamamoto, Elaina Razo, Nicholas P. Krabbe, Mason I. Bliss, Alexander D. Richardson, Morgan A. Einwalter, Andrea M. Weiler, Emily L. Sneed, Kerri B. Fuchs, Xiankun Zeng, Kevin K. Noguchi, Terry K. Morgan, Alexandra J. Alberts, Kathleen M. Antony, Sabrina Kabakov, Karla K. Ausderau, Ellie K. Bohm, Julia C. Pritchard, Rachel V. Spanton, James N. Ver Hoove, Charlene B. Y. Kim, T. Michael Nork, Alex W. Katz, Carol A. Rasmussen, Amy Hartman, Andres Mejia, Puja Basu, Heather A. Simmons, Jens C. Eickhoff, Thomas C. Friedrich, Matthew T. Aliota, Emma L. Mohr, Dawn M. Dudley, David H. O’Connor, Christina M. Newman

**Affiliations:** 1 Department of Pathology and Laboratory Medicine, University of Wisconsin-Madison; Madison, Wisconsin, Unites States of America; 2 Department of Pediatrics, University of Wisconsin-Madison; Madison, Wisconsin, Unites States of America; 3 Wisconsin National Primate Research Center, University of Wisconsin-Madison; Madison, Wisconsin, Unites States of America; 4 United States Army Medical Research Institute of Infectious Diseases; Fort Detrick, Maryland, Unites States of America; 5 Department of Psychiatry, Washington University School of Medicine; St. Louis, Washington, Unites States of America; 6 Department of Pathology, Oregon Health and Science University; Portland, Oregon, Unites States of America; 7 Department of Obstetrics and Gynecology, Oregon Health and Science University; Portland, Oregon, Unites States of America; 8 Department of Obstetrics and Gynecology, University of Wisconsin-Madison; Madison, Wisconsin, Unites States of America; 9 Department of Kinesiology, University of Wisconsin-Madison; Madison, Wisconsin, Unites States of America; 10 Waisman Center, University of Wisconsin-Madison; Madison, Wisconsin, Unites States of America; 11 Department of Veterinary and Biomedical Science, University of Minnesota; St. Paul, Minnesota, Unites States of America; 12 Department of Ophthalmology and Visual Sciences, University of Wisconsin-Madison; Madison, Wisconsin, Unites States of America; 13 Department of Communication Sciences and Disorders, University of Wisconsin-Madison; Madison, Wisconsin, Unites States of America; 14 Department of Biostatistics & Medical Informatics, University of Wisconsin-Madison; Madison, Wisconsin, Unites States of America; 15 Department of Pathobiological Sciences, University of Wisconsin-Madison; Madison, Wisconsin, Unites States of America; University of North Carolina at Chapel Hill School of Medicine, UNITED STATES

## Abstract

In the 2016 Zika virus (ZIKV) pandemic, a previously unrecognized risk of birth defects surfaced in babies whose mothers were infected with Asian-lineage ZIKV during pregnancy. Less is known about the impacts of gestational African-lineage ZIKV infections. Given high human immunodeficiency virus (HIV) burdens in regions where African-lineage ZIKV circulates, we evaluated whether pregnant rhesus macaques infected with simian immunodeficiency virus (SIV) have a higher risk of African-lineage ZIKV-associated birth defects. Remarkably, in both SIV+ and SIV- animals, ZIKV infection early in the first trimester caused a high incidence (78%) of spontaneous pregnancy loss within 20 days. These findings suggest a significant risk for early pregnancy loss associated with African-lineage ZIKV infection and provide the first consistent ZIKV-associated phenotype in macaques for testing medical countermeasures.

## Author summary

The 2016 Zika virus (ZIKV) pandemic revealed a risk of birth defects associated with in-utero exposure to Asian-lineage ZIKV. Less is known about the impacts of African-lineage ZIKV infection during pregnancy, although it has been shown to cause adverse pregnancy outcomes in small animal models. Here we provide evidence that ZIKV infection early in the first trimester caused a high rate (78%) of pregnancy loss in rhesus macaques. These findings suggest a significant risk for early pregnancy loss associated with African-lineage ZIKV infection and provide the first consistent ZIKV-associated phenotype in macaques for testing medical countermeasures.

## Introduction

Zika virus (ZIKV) is a flavivirus discovered in 1947 in Uganda [1]. ZIKV was historically associated with intermittent epidemics throughout Africa, Asia, and Oceania, resulting in mild illness with seemingly few consequences. When the virus emerged in Brazil in 2015, there was an increase in cases of infant microcephaly [2]. This increase in microcephaly and other developmental abnormalities among neonates was ultimately associated with ZIKV exposure in-utero, drawing the attention of the broader scientific community to congenital Zika syndrome (CZS) [3–5]. In the United States, 5–10% of infants with known gestational ZIKV exposure have developmental outcomes consistent with CZS [6]. Although the public health emergency has ended, recent outbreaks in India and evidence of periodic human infections elsewhere suggest ZIKV remains a threat during pregnancy [7–10].

Rhesus macaques (*Macaca mulatta*) have been used to model ZIKV infection during pregnancy using varying gestational time points, strains, doses, and routes of infection. Due to interest in the 2016 ZIKV pandemic, most studies have used Asian-lineage viruses, and in these studies, infection earlier in gestation frequently led to more severe outcomes [11–13]. In a previous meta analysis, we found a fetal demise rate of 26% (n = 50) when using an Asian-lineage strain (PRVABC59) to infect macaques during the first trimester using varying doses and routes (<GD 55) [14]. Across multiple more recent studies using this strain, adverse fetal outcomes remain relatively rare (<10%; n = 21) (S1 Table) [12,13,15–17]. Although <10% is comparable to the rate of adverse outcomes seen in humans, a higher rate of adverse outcomes is needed to make robust comparisons in macaque studies that are inherently limited by small sample sizes.

In 2017, the National Institutes of Health (NIH) launched a study designed to investigate the impact of ZIKV infection on pregnant people coinfected with HIV [18]. The goals of the study were to determine whether infection with one virus increases the risk for infection with the other, the impact of medications that prevent vertical transmission of HIV on ZIKV infection, and the impact of HIV infection on the risk of fetal brain abnormalities observed with in-utero ZIKV infection [18]. To complement this human study, we performed a companion study utilizing rhesus macaques. Unlike human co-infections, a macaque model controls for confounding factors such as the dose, strain, and timing of ZIKV or simian immunodeficiency virus (SIV) infection [19]. SIV-induced disease in macaques manifests similarly to HIV-induced disease in humans, making SIV-infected macaques a useful model for the study of human HIV infection [20]. The same combination ART and pre-exposure prophylaxis (PrEP) therapies prevent and treat both HIV infection in humans and SIV infection in macaques, and treatment during pregnancy is effective in preventing vertical transmission of both HIV and SIV [21,22]. Moreover, frequent sampling of dams throughout pregnancies and neonates after birth enables comprehensive longitudinal monitoring that is difficult to replicate in people.

Areas of high HIV prevalence in sub-Saharan Africa overlap with areas of historical ZIKV infections. Additionally, African-lineage ZIKV strains show greater pathogenicity in mice than Asian-lineage strains and resulted in resorption of all embryos in dams infected with African-lineage ZIKV [23,24]. Therefore, we used an African-lineage virus from Senegal (ZIKV-DAK; strain 41524) in this study to increase the likelihood of adverse pregnancy outcomes. This ZIKV strain was selected based on its availability and limited number of passages in cell culture in order to minimize the likelihood of adaptation to vertebrates [24–26]. Unexpectedly, we found that infection with ZIKV-DAK at GD 30 resulted in early pregnancy loss in 11 of 14 macaques, irrespective of their SIV status. This finding suggests that although ZIKV has not historically been associated with adverse pregnancy and fetal outcomes in Africa, it may represent a previously unrecognized cause of early pregnancy loss. Furthermore, this consistent phenotype provides an opportunity to test ZIKV medical countermeasures during pregnancy.

## Results

### Study design

**Fig 1:**
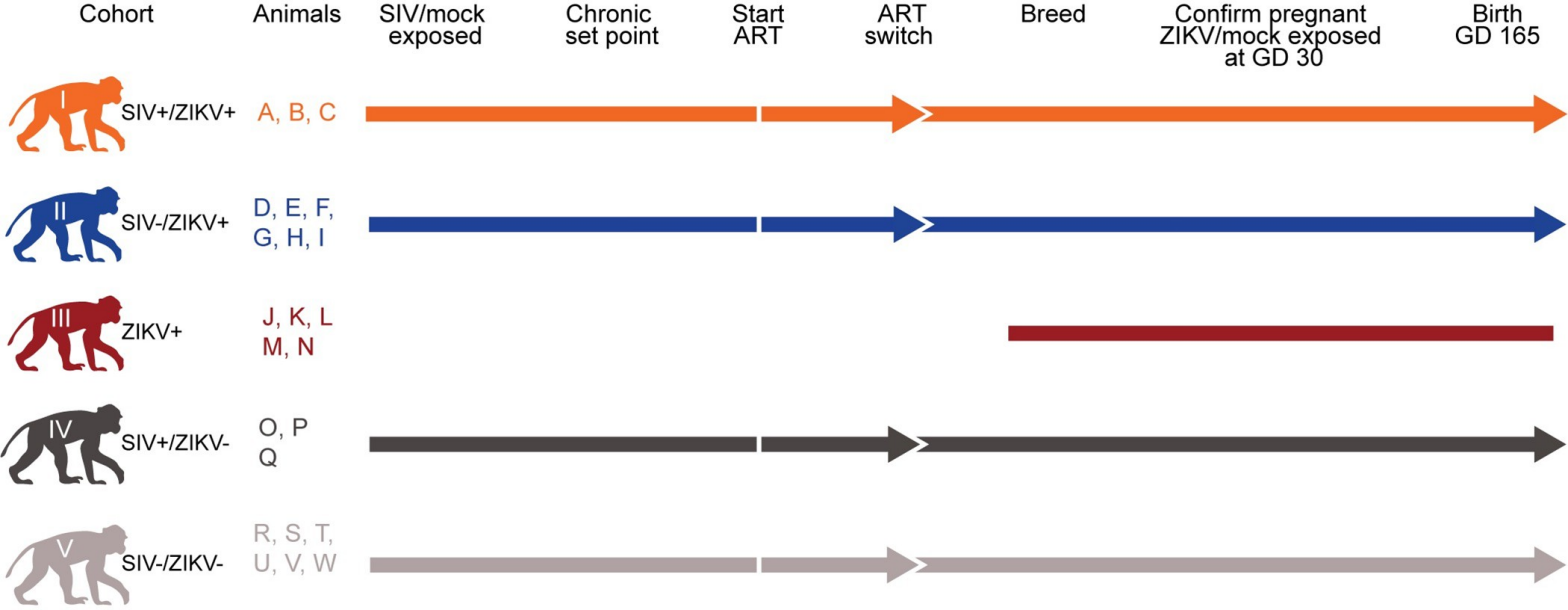
Study Design and cohort definitions. The 23 pregnancies in this study are grouped into Cohorts I-V according to the dam’s viral exposure(s) and antiretroviral therapy (ART) regimens. Once the chronic set point was reached following intrarectal (IR) SIV exposure, the animals began the first ART regimen of tenofovir disoproxil fumarate (TDF), emtricitabine (FTC), and dolutegravir sodium (DTG). Thirty days before breeding, animals were switched to a new ART regimen of TDF/FTC/Raltegravir (RAL). Arrowheads denote a switch in ART regimen. Animals were bred until pregnancy was confirmed by ultrasound. On or around gestational day (GD) 30, animals were exposed subcutaneously (SC) to either ZIKV or PBS (mock). Maternal fetal-interface (MFI) and fetal/embryonic tissues were collected on the date of pregnancy loss or birth (~GD 165). Animals that did not deliver naturally by GD 170 had Cesarean sections (C-sections). Macaque silhouettes were prepared by L. Raasch and are not restricted by copyright.

For this study, twenty-three rhesus macaques were enrolled into one of five Cohorts (Fig 1). Information on the exact timing of infection, treatment with antiretroviral therapy (ART), and pregnancy for all animals is in S2 Table. We reasoned that the impacts of SIV co-infection on ZIKV pathogenesis would be most apparent when ZIKV infection occurs early in pregnancy, so we infected animals at approximately GD 30 (range GD 26–38) which corresponds to GD 49 in human pregnancy [27]. Cohort III was SIV naive and not treated with ART to control for the potential impacts of ART on ZIKV adverse outcomes. All SIV+ animals (Cohorts I and IV) reached a chronic set point before beginning ART and achieved undetectable SIV viremia before subsequent ZIKV/mock exposure (S1 Fig).

### Maternal ZIKV infection

**Fig 2:**
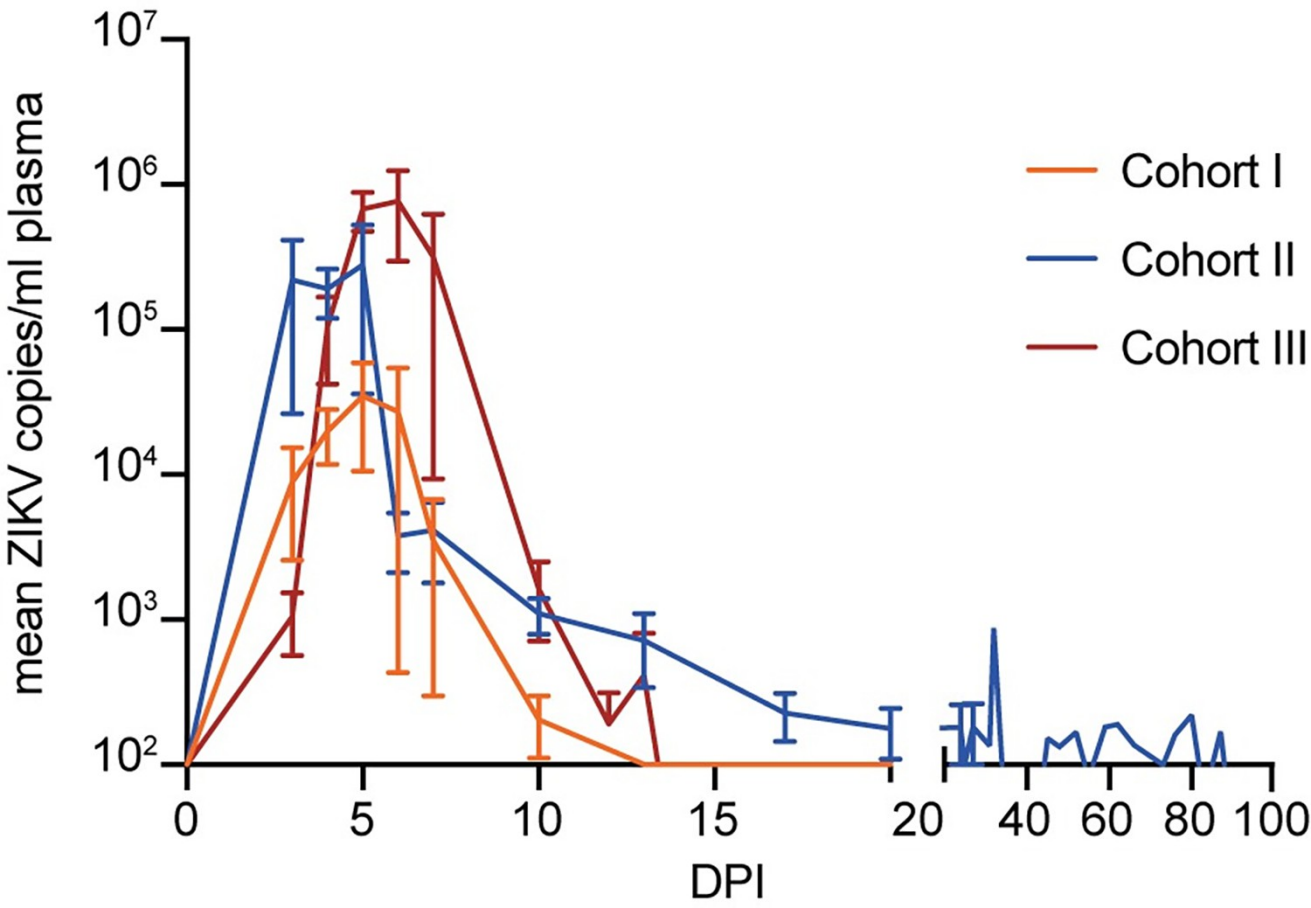
Maternal ZIKV plasma viremia. ZIKV viremia in macaque plasma. Copies of viral RNA were detected by ZIKV-specific RT-qPCR. Mean ZIKV plasma viremia are displayed in orange for Cohort I, blue for Cohort II (SIV-/ZIKV+ +ART), and red for Cohort III (SIV-/ZIKV+). Bars represent the standard error of the mean (SEM).

All fourteen ZIKV-exposed animals (Cohorts I-III) had detectable ZIKV in plasma by 3 days post inoculation (DPI) and reached peak plasma viral load between 4 DPI and 6 DPI (mean 4.7 DPI) (Fig 2)(S2 Fig). ZIKV plasma viremia was resolved for all animals by 20 DPI with the exception of Animal I who had prolonged detection of ZIKV until 132 DPI. All animals, except for the dam in Pregnancy K, had resolved their ZIKV plasma viremia at the time of pregnancy loss (S2 Fig). The dam in Pregnancy K had a viral load of 312.25 copies/ml (S2 Fig), but one day after pregnancy loss the viral load dropped below the limit of quantification. To examine the combined overall magnitude and duration of ZIKV detection in maternal plasma, the area under the curve (AUC) for each dam’s ZIKV plasma viral load was calculated. The AUC values were then compared between Cohorts I through III using a non-parametric equivalent of an ANOVA, the Kruskal-Wallis rank sum test which showed that these values did not significantly differ between cohorts (X^2^ = 5.88, df = 2, p = 0.053). The duration of plasma ZIKV viremia alone was also compared between Cohorts I through III using a Kruskal-Wallis test and although duration tended to be longer for Cohort II, this is likely due to one dam that had prolonged viremia and, overall, it did not differ significantly between cohorts (X^2^ = 3.66, df = 2, p = 0.16). Additionally, the magnitude of peak plasma viremia was compared between Cohorts I through III using a one-way ANOVA and likewise did not differ significantly between cohorts (F(2, 11) = 3.06, p = 0.09). Time from ZIKV exposure to peak ZIKV plasma viral load was also compared between Cohorts I through III using a one-way ANOVA and was not significantly different between groups (F(2,11) = 2.36, p = 0.14). Taken together, ART and SIV infection did not appear to impact subsequent ZIKV infection in terms of maternal plasma viral load dynamics.

### ZIKV-DAK exposure at GD 30 results in a high rate of pregnancy loss

**Fig 3:**
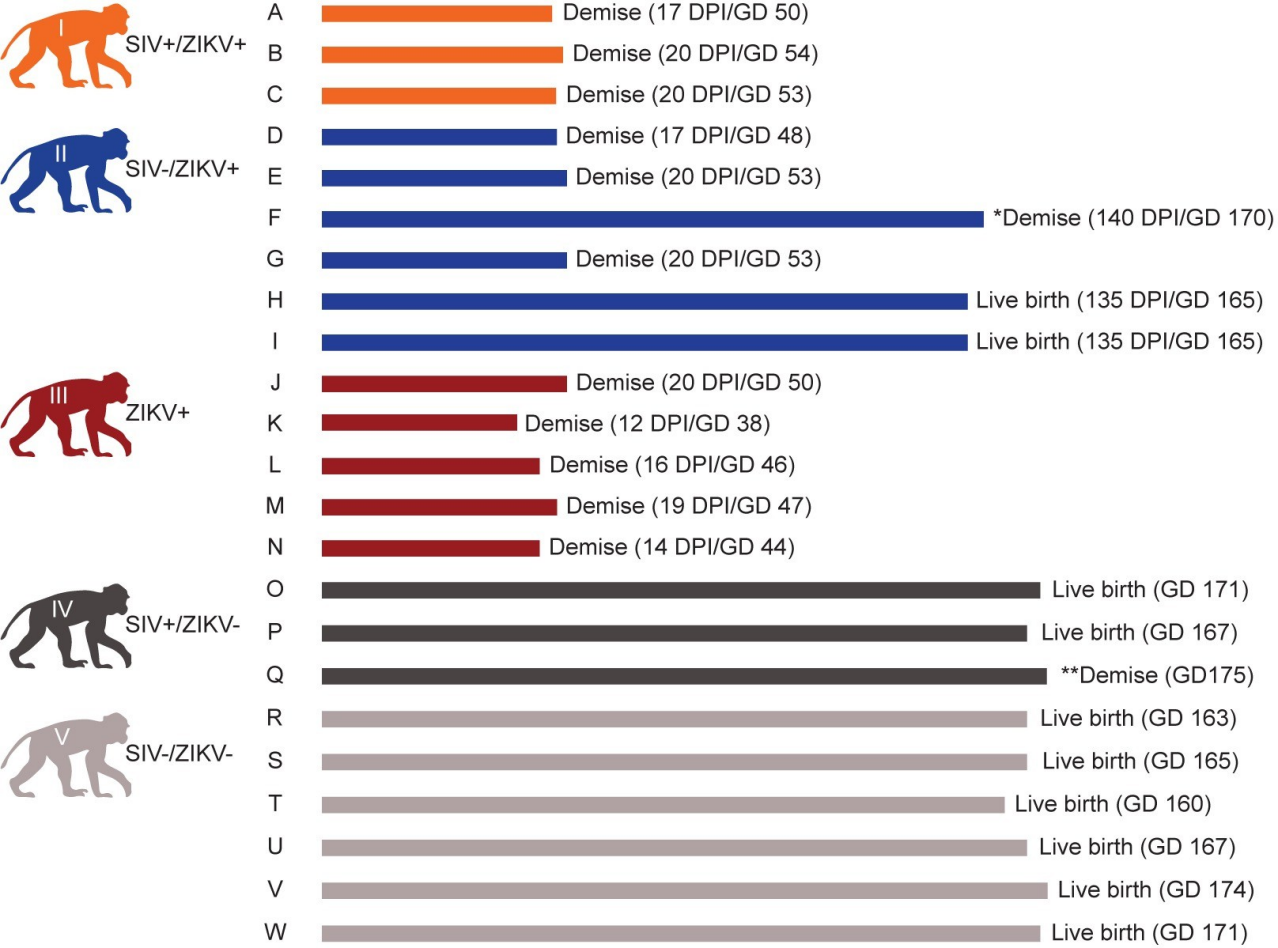
Pregnancy outcomes. *Animal F and fetus died on the day of clinical C-section (GD 170) due to anesthesia complications. **The fetus of Animal Q died 5 days post-due date (GD 175). Macaque silhouettes were prepared by L. Raasch and are not restricted by copyright.

Eleven out of fourteen (78%) ZIKV+ dams experienced pregnancy loss in the first trimester (GD 38–54) (Fig 3, S2 Table. All three dams in Cohort I, three out of six dams in Cohort II, and all five dams in Cohort III experienced this loss at 12–20 days post-ZIKV exposure (Fig 3, S2 Table). SIV infection and ART did not appear to affect the rate of pregnancy loss. A Cohort II full term infant died from complications (cardiac and respiratory arrest) during clinical Cesarean section (C-section), during which the dam also died (Pregnancy F). More details about this case can be found in S3 Table. All animals in the ZIKV naïve cohorts (Cohorts IV and V) maintained viable pregnancies apart from the dam from Pregnancy Q, who experienced a full-term fetal loss around GD 175. This falls within the pregnancy loss rates for non-ZIKV exposed macaques which range from 4–10.9% [14].

### ZIKV is detected throughout the maternal-fetal interface in cases of early pregnancy loss

**Fig 4:**
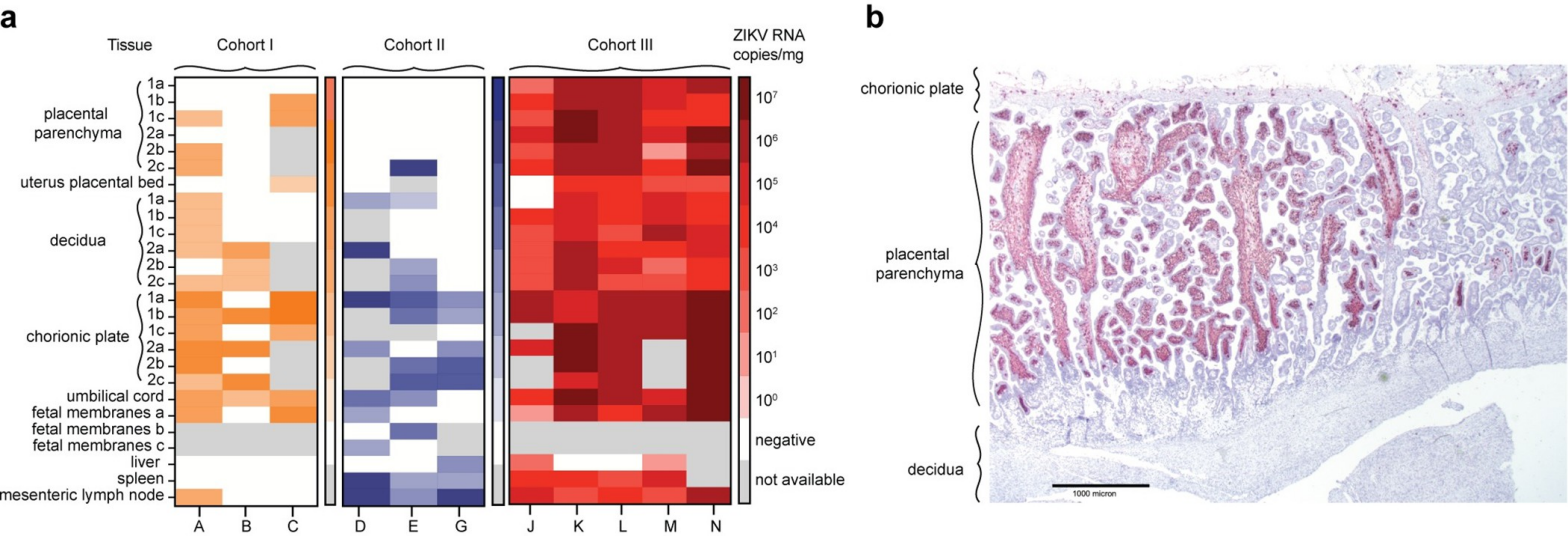
ZIKV was detected in tissues from the maternal-fetal interface (MFI) and dam in all cases of early pregnancy loss. Cohort I (SIV+/ZIKV+ +ART) animals are in orange, Cohort II (SIV-/ZIKV+ +ART) animals are in blue, and Cohort III (SIV-/ZIKV+) animals are in red. (a) ZIKV was detected in MFI and maternal tissues from Cohorts I-III by ZIKV-specific RT-qPCR. The placenta was segmented into cotyledons, labeled as ’a’, ’b’, and ’c’, and a sample was obtained from each layer (decidua, parenchyma, chorionic plate) within every cotyledon. Maternal tissues were taken by biopsy at the time of C-section. (b) Representative image of ZIKV RNA (red staining) detected by in-situ hybridization (ISH) in the first placental disc from a case of pregnancy loss (Pregnancy K). Here, there was marked diffuse villous parenchymal staining extending from the basal plate to the chorionic trophoblastic shell with transmural segmental sparing of villi.

ZIKV nucleic acid was detected in the MFI tissue of all 11 dams that experienced early pregnancy loss and was present in all three placental layers (decidua, placental parenchyma, chorionic plate) (Fig 4A). Nine of 11 dams (Pregnancies A, D, E, G, J, K, L, M, N) across Cohorts I-III had ZIKV RNA detected in a subset of maternal tissues (Fig 4A). Ultrasounds or unsedated fetal doppler were assessed twice weekly for Cohorts I and II (Pregnancies A-G) and daily for Cohort III (Pregnancies J-N) on days 10–21 post-ZIKV infection. Thus, dams from Pregnancies A-G had a potential for miscarriage up to multiple days before detection by ultrasound and subsequent C-section and tissue collection. This delayed collection can lead to greater tissue autolysis and vRNA degradation, making it difficult to compare Pregnancies A-G to Pregnancies J-N. No dams with full term pregnancies had virus detectable in the MFI or maternal tissues.

ZIKV RNA was detected by ISH in the MFI and fetal/embryonic tissues from all cases of early pregnancy loss. In the MFI, RNA was present in the chorionic plate, chorionic villi, and surrounding the chorionic vessels for all 11 cases (S3 Table). The umbilical cord had scattered ISH positivity in five of 11 cases. The ISH signal in MFI tissue was highly concentrated in the chorionic villi where ZIKV RNA was restricted to the villous stroma and absent from the outer syncytiotrophoblast layer. ZIKV RNA was either detected in the stroma of all villi or it was restricted to individual villous structures (S3 Table). ISH staining in the chorionic plate and chorionic vessels was diffuse and much less dense than the staining in the villous stroma (Fig 4B).

### ZIKV is detected in embryonic/fetal tissues and fluids collected from cases of early pregnancy loss

**Fig 5:**
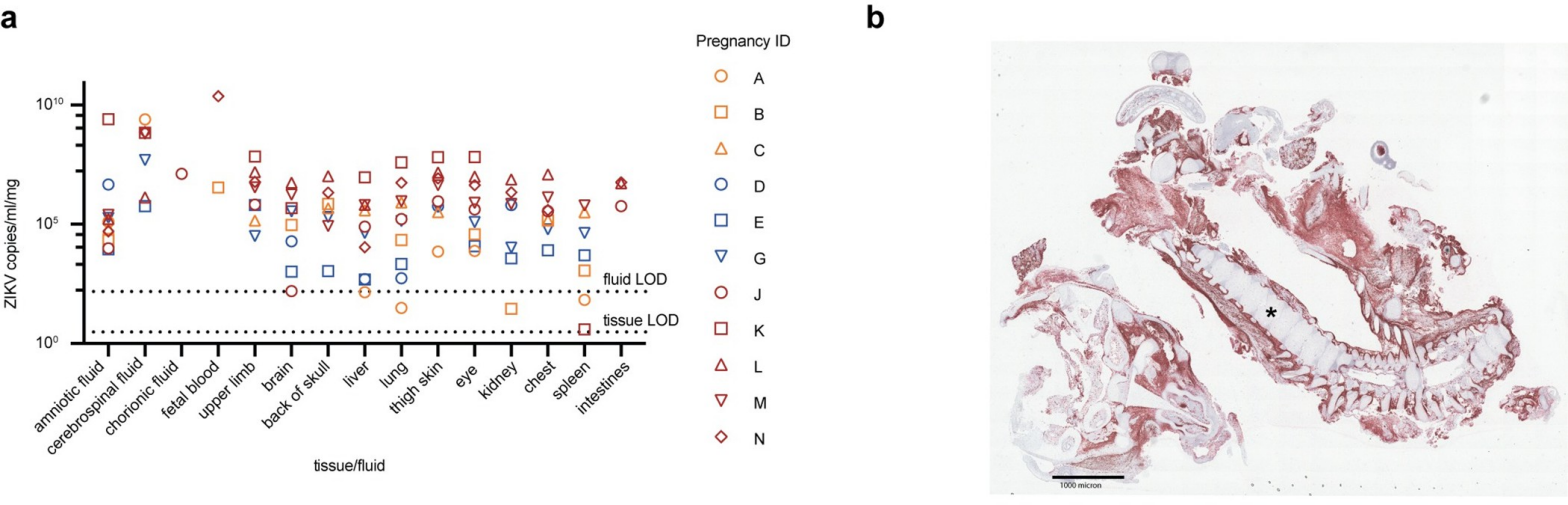
ZIKV was detected in tissues from the fetus/embryo in all cases of early pregnancy loss. Cohort I (SIV+/ZIKV+ +ART) animals are in orange, Cohort II (SIV-/ZIKV+ +ART) animals are in blue, and Cohort III (SIV-/ZIKV+) animals are in red. (a) ZIKV was detected in fetal/embryonic tissues from cases of early pregnancy loss by ZIKV-specific RT-qPCR. LOD denotes the limit of detection. (b) Representative image of ZIKV RNA (red staining) distribution in an embryo from a case of early pregnancy loss (Pregnancy N). Here, ZIKV RNA was detected in the periosteum and musculature of the head and body tissues. The asterisk denotes the vertebral column of the embryo.

ZIKV nucleic acid was detected in embryonic/fetal tissues and fluids from all cases of early pregnancy loss (Fig 5A). The three highest viral loads were detected in the upper limb (6.9x10^7^ copies/mg), eye (6.6x10^7^ copies/mg), and thigh skin (6.6x10^6^ copies/mg) from the embryo of Pregnancy K. This dam had the earliest ZIKV exposure at GD 26 and earliest pregnancy loss at 12 DPI.

ISH signals in the fetal/embryonic tissues indicated ZIKV RNA was present in the following tissues: skeletal muscle, dura mater, periosteum, bone, skin, neuropil, heart, kidney, liver, tongue, intestines, and spinal cord. Each case of fetal loss had a different foci of infection in the aforementioned tissues; however, across all cases, ZIKV RNA was most frequently detected in the skeletal muscle, dura mater, and periosteum (S3 Table). A representative image of fetal ISH staining is seen in Fig 5B.

### MFI and fetal pathology of ZIKV pregnancy loss

Placental lesions were noted in 11 of 12 pregnancies and were most frequently located in the placental parenchyma which includes the villi, intervillous space, chorionic plate, and basal plate. Although there was no specific lesion that was consistent across all cases of pregnancy loss, the most common parenchymal lesions identified included necrosis (Pregnancies C, D, E, F, K, M), hemorrhage (Pregnancies A, B, G, M, N), and neutrophilia (Pregnancies A, D, F, J, K). Other parenchymal lesions that were less frequent included: villous stromal vascular karyorrhexis (VSVK) (Pregnancies C, G, M), chronic histiocytic intervillositis (CHIV) (Pregnancies F, G, J), syncytial trophoblast knots (Pregnancies C, F), and chorionic edema (Pregnancy G). Lesions in the decidua basalis were identified in eight of twelve Pregnancies and include hemosiderosis (Pregnancies A, E, G, L), neutrophilia (Pregnancies D, G, L, N), lymphoplasmacytic infiltration (Pregnancies D, F, L) and necrosis (Pregnancies D, E, L, J). The remaining anatomical sections that were analyzed had identifiable lesions in only three cases: uterus (Pregnancies A, D, L), decidua parietalis (Pregnancies D, E, G), and the umbilical cord (Pregnancies G, J, L). A detailed description of the lesions in these sections can be found in S3 Table. Embryonic, fetal, or infant lesions were also noted in eleven of the twelve cases of pregnancy loss. For cases of early pregnancy loss, the most frequent lesion was diffuse tissue autolysis. Other lesions identified in embryos or fetuses included pulmonary hemorrhage (Pregnancies A, B) and congestion of the cerebral meninges (Pregnancy J). For the pregnancy with a full-term infant loss (Pregnancy F), more extensive lesions were identified and are described in detail in S3 Table.

### Maternal antibody response to ZIKV infection is similar across cohorts

**Fig 6:**
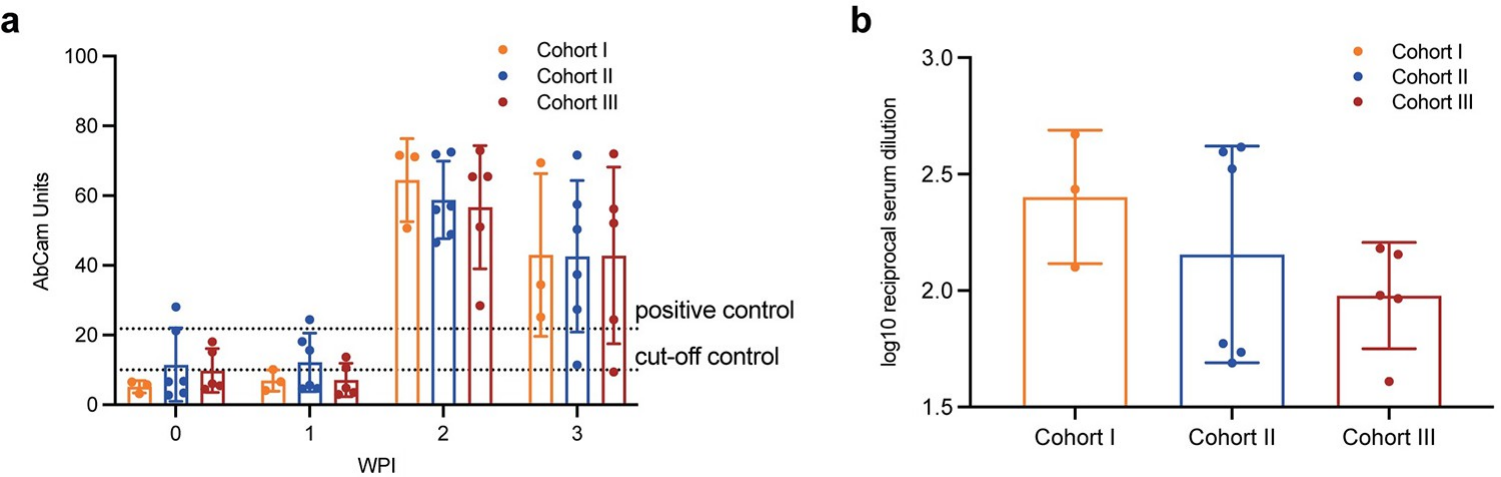
All ZIKV-exposed animals developed robust IgM and neutralizing antibody responses. (a) ELISA determined Anti-ZIKV IgM levels at 0, 1, 2, and 3 weeks post infection (WPI). (b) Neutralizing antibody levels were measured at baseline and 17, 20, or 27 days post infection (DPI) by PRNT 90. Bar graphs depict the mean antibody levels for each cohort at the respective timepoints. Error bars represent the standard deviation.

Maternal ZIKV neutralizing antibodies were examined at 17, 20, or 27 DPI, and by this time all dams had developed robust neutralizing antibody responses to ZIKV. ZIKV-specific immunoglobulin M (IgM) was detected in the maternal serum samples by ELISA. ZIKV-specific IgM reached its peak in the second week post-infection (WPI) in all three cohorts exposed to ZIKV (Cohorts I-III) (Fig 6A). Ninety percent plaque reduction neutralization tests (PRNT_90_) detected protective titers (>1:10) of ZIKV neutralizing antibodies in maternal serum samples from all ZIKV-exposed animals at 17, 20, or 27dpi (Fig 6B). A Pearson’s correlation analysis showed no significant correlation between maternal duration of plasma viremia and PRNT_90_ by 4 WPI (R = -0.39, p = 0.17) (S3 Fig).

### In-utero data

Fetal/embryonic monitoring by ultrasound identified potential risk factors for pregnancy loss in seven of the 11 cases of early pregnancy loss (Pregnancies A, B, E, F, G, J, and N). These risk factors include identification of a thick nuchal fold (Pregnancy B), excess uterine fluid (Pregnancy E), and a retroplacental bleed and clot (Pregnancy G). An additional precursor to fetal demise, fetal hydrops, was noted in multiple pregnancies (A, E, F, J, and N). Comprehensive ultrasound remarks are noted in S4 Table. In-utero fetal measurements including biparietal diameter, head circumference, abdominal circumference, and femur length were taken during ultrasound. A linear mixed-effects regression model with animal specific measurements and an autoregressive correlation structure over WPI did not highlight statistically significant differences between Cohorts II (SIV-/ZIKV+) and IV (SIV+/ZIKV-). However, the comparison of slopes between Cohorts II and IV, and between Cohort IV and V (SIV-/ZIKV-) showed statistically significant differences in in-utero growth (S4 Fig, S5 Table).

### Infant assessments

#### Neonatal and gait development

We defined neonatal neurodevelopment and gait during the first month of life for the live born infants, which included two infants in the ZIKV-exposed group (Cohort II), two in the SIV-exposed group (Cohort IV), and six infants in the control group (Cohort V). There were no live born infants from Cohorts I and III. One infant in Cohort II, from pregnancy H, had worse scores in the developmental area of state control at 7, 14, 21, and 28 days of life compared with all the other infants as evaluated with the Schneider Neonatal Assessment for Primates (SNAP) (S5 Fig). This infant and the other infant from Cohort II scored similarly in other SNAP constructs (S5 Fig). Lower scores in the state control construct means that infant H had difficulty calming themselves and behavioral organization during the testing. Infant H also performed worse than most other infants in visual orientation, visual tracking, and focus (S6 Fig). We found no apparent differences in the majority of gait patterns between infant H and the remaining infants, except for infant H having a wider hind limb base of support potentially indicating difficulty with balance (S7 Fig).

### Hearing and vision development

Longitudinal hearing tests from one month to one year of age found that one infant had no auditory brainstem responses to a low-pitched auditory stimulus at 12 months of age (S8 Fig). This single infant was part of Cohort V (SIV-/ZIKV-), and had normal auditory brainstem responses to higher pitched stimuli (1000 Hz and click stimuli). To confirm that this infant has hearing loss for low pitches, repeat testing will be necessary. Other infants displayed no auditory brainstem responses temporarily earlier in life, followed by normal hearing at 9 months of age. The majority of the no responses occurred at 1 month of age, suggesting that debris within the ear canal may impair responses to auditory stimuli.

The majority of liveborn infants from cohorts II and IV had similar visual function and retinal structure compared to infants from Cohort V, except for one infant. One infant in Cohort II (Pregnancy H) had electroretinography waveform values (B wave amplitude) and visual evoked potentials (P2 wave latency) that did not overlap with Cohort V infants (S9 Fig), suggesting that this infant may have impaired visual function. This infant (Pregnancy H) also had vitreous opacities noted at its 9 and 12 month exams described as “vitreous clumping” by the ophthalmologist (S10 Fig). Retinal structure studies found this infant (Pregnancy H) also had a thicker retina compared with other infants, identified on exams from 1–9 months of age (S11 Fig). No results were available from its 12-month examination for comparison. This infant did not have consistently thicker individual retinal layers, but did have multiple thicker trending individual layers compared to other infants at multiple timepoints.

## Discussion

In its original conception, this study was designed to assess the impact of HIV/SIV on congenital ZIKV infection in macaques. However, early in the study, we observed that animals inoculated with ZIKV-DAK at GD 30 experienced pregnancy loss in the first trimester, regardless of SIV status. This left us unable to assess fetal and neonatal outcomes of coinfection, and because we observed similar rates of pregnancy loss in all ZIKV-exposed cohorts, we ceased enrollment of animals into our SIV+ cohorts (I and IV). This unexpected finding is arguably much more important: we serendipitously developed a model that results in 78% pregnancy loss in macaques and identified a potentially unappreciated risk for early pregnancy loss in women infected with African-lineage ZIKV.

Previously, macaque infection with the same dose of ZIKV at GD 45 did not result in pregnancy loss [28]. The frequent first-trimester demise that we observe with our GD 30 infection model may be influenced by placental development at the time of infection. ZIKV may be more likely to enter the fetal compartment earlier in gestation due to the remodeling of the spiral arteries [29]. In the first weeks of gestation, fetal extravillous cytotrophoblasts infiltrate maternal decidual spiral arteries, increasing uterine artery blood flow to the placenta [30]. Infection during this critical period may allow more virus access into the fetal compartment and increase the risk of demise. This hypothesis is supported by our earliest ZIKV exposure (Pregnancy K) that occurred at GD 26 and resulted in pregnancy loss earliest (12 DPI). The affected embryo also had the three highest tissue viral loads of any pregnancy loss (Fig 5A). However, decidual spiral artery pathology (leukocytoclastic vasculitis), suggesting inflammation at the site of spiral artery remodeling, was diagnosed in only one case of early pregnancy loss (Pregnancy D) (S3 Table).

Within the MFI, viral load data suggests a greater burden of ZIKV RNA in the chorionic plate and placental parenchyma tissue as compared to the decidua, a trend consistent with previous studies that used ZIKV-DAK (Fig 4A) [31]. Viral load data are also consistent with the ISH data from this study, with the strongest ISH signal in the chorionic plate and the stroma of villi in the parenchyma (Fig 4B). Surprisingly, no viral RNA was detected in the decidua by ISH despite detection by RT-qPCR (Fig 4). Although the decidua was negative for vRNA by ISH, pathologic diagnoses commonly associated with infection were identified in the decidua (Fig 4B, S3 Table). Analysis of fetal tissues was hampered by post-mortem autolysis that was present in every case of early demise. Nonetheless, ZIKV RNA was detected in fetal tissues of all early demise cases by RT-qPCR (Fig 5A). Additionally, ISH signals were strong throughout fetal tissues and suggest ZIKV has a tropism for muscle and connective tissues (S3 Table). This supports clinical findings of myalgia and arthralgia as symptoms of flavivirus infections [32].

Congenital Zika syndrome is a complex phenotype that is likely to be influenced by many factors and the rise of CZS in 2015 in the Americas has still not been clearly linked to any one cause. One explanation for CZS prevalence in various regions of the world, could be the lack of surveillance in regions with inadequate access to medical care [33]. Many African communities are medically underserved and pregnancy abnormalities may go unnoticed. Although ZIKV is endemic throughout Africa, seroprevalence varies by country and in many places a significant proportion of the reproductive age population has low serological evidence of prior ZIKV exposure [34–37]. However, prior exposure and therefore immunity remains an important factor to consider when studying the effects of African-lineage ZIKV on pregnancy outcome in these areas. Previous studies have proposed that some flaviviruses cause lesser disease severity in populations of African descent due to protective genetic components [38,39]. However, a protective genetic effect has not been studied in the context of ZIKV infection.

In addition to these hypotheses, we propose that the difference in pregnancy outcomes noted in different regions of the world may be due to African-lineage ZIKV being more pathogenic than the Asian-lineage viruses that impacted the Americas. Previous studies in mice also suggest a similar conclusion: that African-lineage ZIKV could more easily go unnoticed by public health due to a tendency to cause fetal loss rather than birth defects [23,24]. Translating our pregnancy loss rate of 78% in macaques to humans, it is possible that CZS and microcephaly have gone unreported in Africa because ZIKV infections frequently result in early miscarriage that could precede pregnancy detection. Our study in macaques is impactful because the similarities to humans in placental development and immunology make this model particularly translational.

Further studies focusing on mechanisms of the fetal demise phenotype are needed to fully understand the adverse pregnancy outcomes we observed and develop effective countermeasures. It would be useful to conduct cell-specific immunostaining on placenta slides that show positive ZIKV staining as this could identify the infected cell types and suggest a mechanism underlying ZIKV-associated pregnancy loss. Further exploration of the genetic differences between Asian and African lineage ZIKV could provide insight into pathogenic mechanisms. To date, the best-characterized difference is the prM S139N mutation, which was found to be more pathogenic in vitro but was not necessary to cause fetal harm in mice [24]. Our virus stock did not contain this mutation. There is also the possibility that the ZIKV isolate used in this study is not broadly representative of viruses currently circulating throughout sub-Saharan Africa, however, there are few low passage contemporary African-lineage ZIKV isolates available from reference centers to perform comparative analyses [23]. A sustained surveillance effort in African populations will be important to understand if African ZIKV is a looming threat for global health.

There are currently no FDA-approved countermeasures for ZIKV infection (https://www.fda.gov/emergency-preparedness-and-response/mcm-issues/zika-virus-response-updates-fda), in part due to waning ZIKV outbreaks and the absence of a translational pregnancy model that results in consistent outcomes to assess medical countermeasures. Consistent outcomes are needed to make robust comparisons in macaque studies that are inherently limited by small sample sizes. The first-trimester African-lineage ZIKV exposure model described here provides new opportunities for testing therapeutics.

## Materials and methods

### Ethics statement

The macaques used in this study were cared for by the staff at the Wisconsin National Primate Research Center (WNPRC) in accordance with recommendations of the Weatherall report and the principles described in the National Research Council’s Guide for the Care and Use of Laboratory Animals [40]. The University of Wisconsin—Madison, College of Letters and Science and Vice Chancellor for Research and Graduate Education Centers Institutional Animal Care and Use Committee approved the nonhuman primate research under protocol number G006139. The University of Wisconsin—Madison Institutional Biosafety Committee approved this work under protocol number B00000117. Animals were housed in enclosures with the required floor space and fed using a nutritional plan based on recommendations published by the National Research Council. Animals were fed an extruded dry diet with adequate carbohydrates, energy, fat, fiber, mineral, protein, and vitamin content. Diets were supplemented with fruits, vegetables, and other edible objects (e.g., nuts, cereals, seed mixtures, yogurt, peanut butter, popcorn, marshmallows, etc.) to provide variety and to inspire species-specific behaviors such as foraging. To promote psychological well-being, animals were provided with food enrichment, structural enrichment, and/or manipulanda. Environmental enrichment objects were selected to minimize the chances of pathogen transmission from one animal to another and from animals to care staff. While on the study, all animals were evaluated by trained animal care staff at least twice daily for signs of pain, distress, and illness by observing appetite, stool quality, activity level, and physical condition. Animals exhibiting abnormal presentation for any of these clinical parameters were provided appropriate care by attending veterinarians. Before all minor/brief experimental procedures, macaques were sedated using ketamine anesthesia and regularly monitored until fully recovered.

### Study design

Twenty-three female rhesus macaques (*Macaca mulatta)* were divided into five cohorts denoted Cohort I through Cohort V (S2 Table). Animals were enrolled into cohorts at random, based on lack of previous flavivirus exposure, and having never rejected a previous infant. Animals were not age matched across cohorts (Cohort I mean = 9 years; Cohort II mean = 10.1 years; Cohort III mean = 7.4 years; Cohort IV mean = 8 years; Cohort V mean = 13.1 years) (S2 Table). Although all animals were within the captive breeding age range for rhesus macaques (>3 years), Cohort III dams were younger on average than the dams in Cohort V (F (3) = 3.397, p = 0.039), but were not significantly younger than the animals in the other cohorts [41]. Cohorts I (SIV+/ZIKV+ +ART), II (SIV-/ZIKV+ +ART), IV (SIV+/ZIKV- +ART), and V (SIV-/ZIKV- +ART) were exposed to 300 TCID_50_ SIV-mac239 (SIV+) or mock (SIV-) with 1xPBS intrarectally (IR). The dam from Pregnancy O (Cohort IV) was not successfully infected with SIV. Thus, this animal was re-exposed to 500TCID_50_ SIV-mac239 intravenously 21 days after IR exposure. These four cohorts were treated once daily with injectable combination ART (+ART) consisting of tenofovir disoproxil fumarate (TDF), emtricitabine (FTC), and dolutegravir sodium (DTG) (see **Antiretroviral therapy**). Once treated SIV+ animals controlled viremia under the limit of quantification of our in-house SIV RT-qPCR assay (<200 copies/ml plasma, see **Viral RNA quantification by RT-qPCR**), animals had their combination ART switched to an injectable combination of ART of TDF and FTC with two oral doses of Raltegravir (RAL, 100mg/dose) for 30 days before and throughout breeding (see **Antiretroviral therapy**). All five cohorts underwent timed breeding until pregnancy was confirmed by ultrasound. Animals maintained combination ART (TDF/FTC/RAL) throughout pregnancy. At approximately GD 30, animals in Cohorts I, II, and III, were subcutaneously (SC) exposed to 1x10^4^ plaque forming units (PFU)/ml of African-lineage ZIKV (ZIKV+), while Cohorts IV and V were SC exposed to 1xPBS (ZIKV-). Animals were enrolled in Cohort III (ZIKV+ -ART) to confirm that adverse pregnancy outcomes in Cohort I (SIV+/ZIKV+ +ART) and Cohort II (SIV-/ZIKV+ +ART) were the result of ZIKV exposure and not additive impact from ART treatment. All pregnancies were monitored throughout the study with weekly ultrasounds and plasma vRNA load quantification of ZIKV and SIV where appropriate. Pregnancies were allowed to go to term and natural delivery; however, a C-section was performed in the event of an overdue pregnancy (GD 175) (Pregnancies F, O, Q, and W) or demise (no detection of fetal/embryonic heartbeat). In cases of demise, the C-section was followed by a fetal or embryonic necropsy, maternal biopsies, and MFI tissue collection. Following full-term delivery, infants were dam-reared.

### Antiretroviral therapy

Animals in Cohorts I, II, IV, and V were treated daily with an injectable combination ART of sterile-filtered TDF (final concentration 5.1mg/ml), FTC (final concentration 50mg/ml), and DTG (final concentration 2.5mg/ml) in the commercially-available solubility vehicle Kleptose (Roquette, Gurnee, IL). ART drugs were sourced from Hangzhou APIChem Technology Co., Ltd. (Hangzhou, Zhejiang, China) and were confirmed by mass-spectrophotometry at the University of Wisconsin-Madison Genetics and Biotechnology Center. This combination of ART drugs, which includes two nucleoside reverse transcriptase inhibitors (TDF and FTC) and an integrase inhibitor (DTG), has been previously shown to control SIV infection in macaques when provided as a combination injectable at a dose of 1ml/kg [42,43]. Beginning 30 days before breeding and then continuing throughout pregnancy, animals were given oral doses of raltegravir (RAL, 100mg/dose) twice daily alongside a modified daily injection containing TDF and FTC (5.1mg/ml and 50mg/ml, respectively). The integrase inhibitor RAL was used in place of DTG at this study stage due to the potential association of DTG with neural tube defects in human infants when used during pregnancy [44]. Following birth, animals transitioned from oral RAL/double-combination injectable back to triple-combination injectable for continued maintenance of SIV infections. Mock-SIV animals stopped ART after the births of their infants.

### Ultrasonography and fetal monitoring

Ultrasounds and unsedated fetal doppler were conducted weekly (Cohorts I, II, IV, and V) to observe the growth and viability of the fetus and to obtain measurements including fetal femur length (FL), biparietal diameter (BPD), head circumference (HC), and heart rate as previously described [12,45]. Mean growth measurements were plotted against mean measurements and standard deviations from specific gestational days collected from rhesus macaques [46]. Comparison of experimental growth parameters with the established growth curves allowed extrapolation of actual gestational age versus predicted gestational age [12]. The standard growth curve was extrapolated to contextualize measurements collected before GD 50. For Cohort III, unsedated fetal dopplers were performed more frequently (daily from 10–21 days post-ZIKV infection) to confirm viability.

### ZIKV infection

Zika virus strain Zika virus/A.africanus-tc/Senegal/1984/DAKAR 41524 (ZIKV-DAK; GenBank: KX601166, SRR7879856) was originally isolated from *Aedes luteocephalus* mosquitoes in Senegal in 1984. One round of amplification on *Aedes pseudocutellaris* cells, followed by amplification on C6/36 cells, followed by two rounds of amplification on Vero cells, was performed by BEI Resources (Manassas, VA) to create the stock [24]. Once obtained, an additional expansion was performed on C6/36 cells. This virus stock was not contaminated with the insect-specific virus found in the original BEI isolate [24]. Stocks used to infect the animals were prepared from three different passages and sequencing showed the stock viruses to be identical at the consensus level. No minor variants were present at >10% in any of the stocks. For virus challenges, ZIKV-DAK stock was diluted to 1x10^4^ PFU in 1ml of 1x phosphate buffered saline (1x PBS) and delivered to each dam SC over the cranial dorsum via a 1ml luer lock syringe.

### SIV infection

Simian immunodeficiency virus (SIV-mac239, Genbank: M33262) stock was produced from two plasmids acquired from the AIDS Reagent Resource. Plasmids were ligated and transfected in E6 Vero cells. Cell-derived supernatant was then used to infect cultured macaque CD8- depleted peripheral blood mononuclear cells (PBMC), which were then monitored for virus production. The supernatant was harvested at peak virus production. SIV-mac239 was initially used to intra-rectally (IR) expose all animals in Cohorts I and IV (A, B, C, P/Q, and O) at a dose of 300 TCID_50_. Following initial IR exposure, Animal O was found to be uninfected and was subsequently re-exposed intravenously with 500 TCID_50_ with the same virus stock 21 days later. All virus stock dilutions were made in sterile-filtered 1x PBS and administered in a 1ml syringe.

### Blood and body fluids monitoring

Blood samples were collected for isolation of plasma and PBMC from dams prior to SIV infection on days -1 and 0, post-SIV infection on days 7, 13, 14, 16, weekly through 4 weeks post-infection, and twice weekly until ZIKV infection. Blood samples, urine, and saliva were collected on days -1, 0, 3–7, 10, 14, post-ZIKV challenge, and then twice weekly until 28 DPI or until ZIKV was undetectable in blood plasma by RT-qPCR. Samples were then collected weekly until birth.

### Viral RNA isolation from plasma, urine, and saliva

Plasma and PBMC were isolated from EDTA-treated whole blood by layering blood on top of ficoll in a 1:1 ratio and performing centrifugation at 1860x rcf for 30 minutes with no brake. Plasma and PBMC were extracted and transferred into separate sterile tubes. R10 medium warmed at 37 degrees Celsius was added to PBMC before a second centrifugation of both tubes at 670 x rcf for 8 minutes. Before treatment, media was removed from PBMC with 1x Ammonium-Chloride-Potassium (ACK) lysing buffer for 5 minutes to remove red blood cells. An equal amount of R10 medium was added to quench the reaction before another centrifugation at 670 x rcf for 8 minutes. Supernatant was removed before freezing down of cells in CryoStor CS10 medium (BioLife Solutions, Inc., Bothell, WA) for long-term storage in liquid nitrogen freezers. Serum was obtained from clot activator tubes by centrifugation at 670 x rcf for 8 minutes or from serum separation tubes (SST) at 1400 x rcf for 15 minutes. Urine was passively collected from the bottom of animals’ housing, centrifuged for 5 minutes at 500 x rcf to pellet debris, and 270ul was added into 30ul dimethyl sulfoxide (DMSO) followed by slow freezing. Saliva swabs were obtained and put into 500ul viral transport media (VTM) consisting of tissue culture medium 199 supplemented with 0.5% FBS and 1% antibiotic/antimycotic. Tubes with swabs were vortexed and centrifuged at 500 x rcf for 5 minutes. Viral RNA (vRNA) was extracted from 300uL plasma, 300uL saliva+VTM, or 300ul urine+DMSO using the Maxwell RSC Viral Total Nucleic Acid Purification Kit on the Maxwell 48 RSC instrument (Promega, Madison, WI).

### Maternal, fetal, and maternal-fetal interface tissue (MFI) collection from first-trimester pregnancy losses

Following early pregnancy loss, fetal, maternal, and MFI tissues were harvested by board certified veterinary pathologists at the WNPRC. Recovered MFI tissues for pathological evaluation included three sections from each placental disc, amniotic/chorionic membrane from each placental disc, decidua from each placental disc, and one section from the decidua parietalis (fetal membranes), umbilical cord, and uterus/placental bed. One section of each of the following maternal or fetal tissues was also collected: maternal liver, maternal spleen, mesenteric lymph node (LN), fetal liver, fetal intestine, fetal lung, fetal kidney, fetal brain, fetal skin/muscle from thigh, fetal eye, fetal spleen, fetal upper limb, fetal chest, and fetal skull with brain. Two samples from each tissue section were collected and stored in either 750ul VTM or 1mL RNAlater for vRNA assessment and future analysis. Tissues in VTM were frozen immediately after collection and stored at -80°C. Tissues in RNAlater were refrigerated for 24 hours at 4°C, after which RNAlater was aspirated off, and the tissues were stored at -80°C prior to vRNA isolation.

### SIV RNA quantification by RT-qPCR

Viral RNA was quantified using an RT-qPCR assay based on the one developed by Cline et al.[47]. RNA was reverse transcribed and amplified using the TaqMan Fast Virus 1-Step Master Mix RT-qPCR kit (Invitrogen) on the LightCycler 480 instrument (Roche, Indianapolis, IN), and quantified by interpolation onto a standard curve made up of serial ten-fold dilutions of in vitro transcribed RNA. RNA for this standard curve was transcribed from the p239gag_Lifson plasmid kindly provided by Dr. Jeffrey Lifson, NCI/Leidos. The final reaction mixtures contained 150 ng random primers (Promega, Madison, WI), 600 nM each primer, and 100 nM probe. Primer and probe sequences are as follows: forward primer: 5’-GTCTGCGTCATPTGGTGCATTC-3,

reverse primer:5′-CACTAGKTGTCTCTGCACTATPTGTTTTG-3′ and

probe:5′-6-carboxyfluorescein-CTTCPTCAGTKTGTTTCACTTTCTCTTCTGCG-BHQ1-3’.

The reactions cycled with the following conditions: 50°C for 5 minutes, 95°C for 20 seconds followed by 50 cycles of 95°C for 15 seconds and 62°C for 1 min. The limit of detection of this assay is 200 copies/ml.

### ZIKV RNA isolation from tissue samples

Isolation of RNA from tissue samples was performed using a modification of the method described by Hansen, et al.[48]. Up to 200mg of tissue was disrupted in TRIzol Reagent (Thermo Fisher Scientific, Waltham, MA) with stainless steel beads (2x5 mm) using a TissueLyser (Qiagen, Germantown, MD) for three minutes at 25 r/s twice. Following homogenization, samples in TRIzol were separated using bromo-chloro-propane (Sigma, St. Louis, MO). The aqueous phase was collected into a new tube and glycogen was added as a carrier. The samples were washed in isopropanol and ethanol-precipitated overnight at -20°C. RNA was then fully re-suspended in 5 mM Tris pH 8.0.

### ZIKV RNA quantification by RT-qPCR

Viral RNA was quantified using a highly sensitive RT-qPCR assay based on the one developed by Lanciotti et al.[49], though the primers were modified to accommodate both Asian and African lineage ZIKV lineages. Each sample was run in duplicate. RNA was reverse transcribed and amplified using the TaqMan Fast Virus 1-Step Master Mix RT-qPCR kit (Invitrogen) on a LightCycler 480 or LC96 instrument (Roche, Indianapolis, IN), and quantified by interpolation onto a standard curve made up of serial tenfold dilutions of in-vitro transcribed RNA. RNA for this standard curve was transcribed from a plasmid containing an 800 base pair region of the ZIKV genome targeted by the RT-qPCR assay. The final reaction mixtures contained 150 ng random primers (Promega, Madison, WI), 600 nM each primer and 100 nM probe. Primer and probe sequences are as follows:

forward primer: 5’-CGYTGCCCAACACAAGG-3’

reverse primer: 5′-CCACYAAYGTTCTTTTGCABACAT-3′

and probe: 5′-6-carboxyfluorescein-AGCCTACCTTGAYAAGCARTCAGACACYCAA-BHQ1-3’.

The reactions cycled with the following conditions: 50°C for 5 minutes, 95°C for 20 seconds followed by 50 cycles of 95°C for 15 seconds, and 60°C for 1 min. The limit of detection of this assay in body fluids is 150 copies/ml and 3 copies/mg in tissues.

### IgM ELISA

An IgM ELISA was performed on serum samples collected on days 0, 7, 13, and 21 following ZIKV infection. Samples were run in triplicate using the AbCam anti-Zika virus IgM micro-capture ELISA kit protocol according to the manufacturer’s instructions (cat# ab213327, Abcam Inc., Cambridge, UK). Briefly, samples were thawed to room temperature, added to an anti-human IgM-coated microplate tray (μ capture), and incubated. Zika virus conjugate+HRP was added, followed by TMB substrate solution (3, 3’, 5, 5’-tetramethylbenzidine < 0.1%), and stop solution (sulphuric acid, 0.2 mol/L). The plate absorbance was read at dual wavelengths of 450nm and 600nm within 30 minutes of adding the stop solution, and the IgM concentration was measured in the calculated Abcam units (AU) relative to the kit cut-off control. To calculate the AU, the 600nm well data were first subtracted from the 450nm well data. Because multiple samples were run for each animal at each DPI, the average of the numbers was calculated, multiplied by ten, and divided by the absorbance of the cut-off control to get a single AU value per sample. Samples were considered positive if they were above 10 AU and negative if they were below 10 AU.

### Plaque reduction neutralization test

Titers of ZIKV neutralizing antibodies (nAb) were determined for days 0, 17, 20, or 27 days post-ZIKV infection using PRNT on Vero cells (ATCC #CCL-81) with a cutoff value of 90% (PRNT_90_) [50]. Briefly, ZIKV-DAK was mixed with serial 2-fold dilutions of serum for 1 hour at 37°C prior to being added to Vero cells. Neutralization curves were generated in GraphPad Prism (San Diego, CA) and the resulting data were analyzed by nonlinear regression to estimate the log_10_ reciprocal serum dilution required to inhibit 90% infection of Vero cell culture [50,51].

### *In situ* hybridization (ISH)

ISH probes against the ZIKV genome were commercially purchased (cat# 468361, Advanced Cell Diagnostics, Newark, CA). ISH was performed using the RNAscope Red 2.5 kit (cat# 322350, Advanced Cell Diagnostics, Newark, CA) according to the manufacturer’s protocol. After deparaffinization with xylene, a series of ethanol washes, and peroxidase blocking, sections were heated with the antigen retrieval buffer and then digested by proteinase. Sections were then exposed to the ISH target probe and incubated at 40°C in a hybridization oven for two-hours. After rinsing, the ISH signal was amplified using the provided pre-amplifier followed by the amplifier-containing labeled probe binding sites, and developed with a Fast Red chromogenic substrate for 10 minutes at room temperature. Sections were then stained with hematoxylin, air-dried, and mounted.

### Statistical analyses

We compared the ages of the dams between cohorts using an ANOVA and the post-hoc analysis, Tukey’s honestly significant difference (Tukey’s HSD), in R Studio v. 1.4.1717. We defined peak plasma viremia as the highest ZIKV plasma viremia detected for each dam in Cohorts I-III. Plasma viremia duration was defined for these animals as the last time point a dam had ZIKV detected in plasma by RT-qPCR above the limit of quantification of the assay. All plasma viremia analyses were done using raw, untransformed data. Overall plasma ZIKV RNA loads were calculated for all ZIKV-infected dams (Cohorts I-III) using the trapezoidal method to calculate AUC in R Studio v. 1.4.1717. AUC values were then compared between Cohorts I-III using a Kruskall-Wallis rank sum test. Peak plasma ZIKV RNA loads, as well as the duration of positive ZIKV RNA detection, were also compared between Cohorts I-III using a Kruskall-Wallis rank sum test (duration) and one-way ANOVA (peak plasma viremia) using R Studio v. 1.4.1717. The time to peak plasma ZIKV RNA load was also compared between dams in Cohorts I-III. Time to peak was analyzed using a one-way ANOVA to compare between Cohorts. For all analyses of plasma ZIKV RNA loads, p<0.05 was used to define statistical significance. The duration of plasma viremia and PRNT_90_ value at 17, 20, or 27 DPI were assessed for a correlation using Pearson’s correlation analysis in the package ggpubr version 4.1.2 in R Studio v. 1.4.1717. In-utero growth trajectories of abdominal circumference (AC), biparietal diameter (BPD), femur length (FL), and head circumference (HC) were quantified by fitting a linear mixed-effects regression model with animal-specific random effects and an autoregressive correlation structure over time, in this case, weeks post-infection (WPI) using SAS version 9.4 (SAS Institute, Cary NC). Since the growth trajectories were non-linear, a log transformation for each outcome was used. Growth trajectories were compared between Cohorts I, IV, and V by comparing the corresponding slopes (S5 Table) and graphs were generated using R software v. 4.1.0 (R Foundation for Statistical Computing) (S4 Fig). No statistical analyses of infant development, vision, and hearing tests were performed due to small group sizes.

### Infant developmental tests

The Schneider Neonatal Assessment for Primates (SNAP) was used to assess the neurodevelopmental areas of interest (Orientation, Motor maturity and activity, Sensory responsiveness, and State control). This neonatal test is well validated and has previously been used to define neonatal development of prenatally ZIKV-exposed infants [16]. The Catwalk XT version 10.6 was modified for infant rhesus macaques and used to assess gait development, as described previously [31,52]. The SNAP was administered at 7, 14, 21, and 28 (+/- 2) days of life, and the Catwalk was administered on 14, 21, and 28 days of life.

### Infant vision and hearing tests

Infants were anesthetized for eye exams performed by a human ophthalmologist with retinal fellowship training (M. Nork). Slit-lamp biomicroscopy and indirect ophthalmoscopy were performed after pupillary dilation. To evaluate visual function, standard visual electrodiagnostic procedures including a full-field electroretinogram (ERG) and the cortical-derived visual evoked potential (VEP) were performed as previously described [13]. To define retinal layer structure, spectral-domain optical coherence tomography (OCT) was performed as previously described [13]. Auditory brainstem response (ABR) testing was completed, which measures brainstem evoked potentials generated by a brief click, 500 Hz stimulus, or 1000 Hz stimulus, as previously described. The presence or absence of a Wave IV response was recorded for each decibel level and stimulus [13]. The presence or absence of a Wave IV response was recorded for each decibel level and stimulus.

## Supporting information

S1 Fig Maternal SIV plasma viremia. SIV viremia in macaque plasma before and after ART regimen. Copies of viral RNA were determined by SIV-specific RT-qPCR. Cohort I (SIV+/ZIKV+ +ART) pregnancies are in orange, Cohort IV (SIV+/ZIKV- +ART) pregnancies are in dark gray. The dotted line at 90 DPI indicates when animals started the 1x daily injectable combination ART regimen (TDF/FTC/DTG). Pregnancies P and Q are from the same dam and thus have the same SIV plot lines depicted here.
(TIF)

S2 Fig Maternal ZIKV plasma viremia in relation to adverse pregnancy outcomes. Copies of viral RNA were determined by ZIKV-specific RT-qPCR. Gray dotted lines denote the timing of pregnancy loss and the corresponding pregnancy ID(s) is/are listed above the line. Viremia corresponding to animals from (a) Cohort I are in orange, (b) Cohort II are in blue, and (c) Cohort III are in red. Plasma viremia was below the limit of quantification (LOQ) for all animals at the time of pregnancy loss except for Pregnancy K. This viral load dropped below the LOQ one day after pregnancy loss.
(TIF)

S3 Fig Absence of correlation between maternal plasma viremia duration and PRNT_90_ at 17, 20, or 27 days post infection. A Pearson’s correlation analysis showed no significant correlation between maternal duration of plasma viremia and PRNT_90_ at 17, 20, or 27 DPI (R = -0.39, p = 0.17)
(TIF)

S4 Fig In-utero growth trajectories of Abdominal circumference (AC), Biparietal Diameter (BPD), Femur length (FL), and Head circumference (HC) compared between Cohorts II, IV, and V. Slopes for each Cohort were determined using a linear mixed effects regression model with animal specific measurements and an autoregressive correlation structure over WPI. (TIF)

S5 Fig Neurodevelopmental testing was measured by the SNAP at 7, 14, 21, and 28 days of life. The SNAP is composed of 4 main constructs: (a) Orientation, (b) Motor maturity and activity, (c) Sensory responsiveness, and (d) State control. Cohort II (SIV-/ZIKV+ +ART) infants are in blue, Cohort IV (SIV+/ZIKV- +ART) infants are in light gray, and Cohort V (SIV-/ZIKV- +ART) infants are in dark gray. Individual infants are represented by symbols that correspond to their Pregnancy ID.
(TIF)

S6 Fig SNAP Orientation construct was separated by sensory modality and task into 4 main subgroups to see where differences may be occurring. The SNAP Orientation subgroups consist of A) Visual orientation, B) Visual tracking, C) Focus, and D) Auditory orientation. Cohort II (SIV-/ZIKV+ +ART) infants are in blue, Cohort IV (SIV+/ZIKV- +ART) infants are in light gray, and Cohort V (SIV-/ZIKV- +ART) infants are in dark gray. Individual infants are represented by symbols that correspond to their Pregnancy ID.
(TIF)

S7 Fig Infant locomotion was measured at 14, 21, and 28 days of life using the Noldus Catwalk. (a) Visual representation of the gait variables included mature walking pattern (where the contralateral limbs are moving through the swing and stance phase close together in timing), speed (how fast the infant walked across the catwalk), base of support (distance between right and left limbs), and duty cycle (percent of time the infant is standing on the walkway). (b) Percent of time infants use a mature walking pattern. (c) Average speed across runs, (d) Base of support for the front and hind limbs, (e) Duty cycle time the infant was standing on each limb. Cohort II (SIV-/ZIKV+ +ART) infants are in blue, Cohort IV (SIV+/ZIKV- +ART) infants are in light gray, and Cohort V (SIV-/ZIKV- +ART) infants are in dark gray. Individual infants are represented by symbols that correspond to their Pregnancy ID.
(TIF)

S8 Fig Hearing: longitudinal auditory brainstem responses. Infant macaques were tested via auditory brainstem response to click, 1000 Hz, and 500 Hz stimuli. The presence or absence of a Wave IV response for each ear was assessed and summarized for each animal at 1, 3, 6, 9, and 12 months of age. Not all the animals received testing at 12 months of age because some were not 12 months old yet. Infants with normal hearing are expected to have a score of “0” with both ears having a Wave IV auditory brainstem response at the lowest intensity level tested. Cohort II (SIV-/ZIKV+ +ART) infants are in blue, Cohort IV (SIV+/ZIKV- +ART) infants are in light gray, and Cohort V (SIV-/ZIKV- +ART) infants are in dark gray. Individual infants are represented by symbols that correspond to their Pregnancy ID.
(TIF)

S9 Fig Visual electrophysiology. Visual function studies comprising photopic single flash electroretinograms (ERGs) and visual evoked potentials (VEPs). (I) Schematic representing the origins of the A and B waves within an ERG and the N70 and P90 waves within a VEP for each age group, aligned with the ages in part II. These representative waveforms were created from the averages of all the traces for the controls at each time point. (II) ERG A- and B-wave components were measured at 1, 3, 6, 9, and 12 months of age (A-J). The right and left eyes are plotted as individual data points. VEP N70- and P90-wave components were measured at 1, 3, 6, 9, and 12 months of age (K-T). The right and left hemispheres are plotted as individual data points. Cohort II (SIV-/ZIKV+ +ART) infants are in blue, Cohort IV (SIV+/ZIKV- +ART) infants are in light gray, and Cohort V (SIV-/ZIKV- +ART) infants are in dark gray. Individual infants are represented by symbols that correspond to their Pregnancy ID.
(TIF)

S10 Fig Retinal and vitreous images of the infant from Pregnancy H. Optical coherence tomography images of the infant from Pregnancy H at 9 at 12 months of age. Vitreous opacities, or “clumping”, is seen in both right and left eyes, indicated by the white arrows. The retinal layers are denoted with a black bracket. The green arrow shows the relation of the retinal section shown in relation to the fovea.
(TIF)

S11 Fig Optical coherence tomography (OCT): retinal layer thicknesses. Retinal layer thicknesses were measured by optical coherence tomography (OCT) in infants at 1, 3, 6, 9, and 12 months of age. Total retinal thickness, choroidal thickness, and the thickness of individual retinal layers (ganglion cell layer, inner nuclear layer, inner plexiform layer, outer nuclear layer, outer plexiform layer, photoreceptor inner segment, photoreceptor outer segment, retinal nerve fiber layer) were determined by segmentation. Cohort II (SIV-/ZIKV+ +ART) infants are in blue, Cohort IV (SIV+/ZIKV- +ART) infants are in light gray, and Cohort V (SIV-/ZIKV- +ART) infants are in dark gray. Individual infants are represented by symbols that correspond to their Pregnancy ID.
(TIFF)

S1 Table Demise rate of Asian-lineage pregnancy studies in macaques. Across multiple studies the demise rate was <10% when animals were exposed to an Asian-lineage strain of Zika virus, PRVABC59.
(XLSX)

S2 Table Correspondent demographic information by Cohort and Pregnancy ID throughout the study period. Age and weight presented correspond to the measures taken at the ZIKV challenge date. GD denotes gestational day and DPI denotes days post infection. Animals with pregnancies that made it to near-term delivered naturally (N) or by Cesarean section (C). In all cases of pregnancy loss the fetus/embryo was extracted by Cesarean section (C).
(XLSX)

S3 Table Histological diagnosis and localization of ZIKV RNA in placental and embryonic/fetal tissue from cases of early pregnancy loss (CSV). Lesions are graded on a scale of 0–4: 0 = no inflammation/pathology/necrosis; 1 = minimal; 2 = mild; 3 = moderate; 4 = severe. Localization of ZIKV RNA was determined by in-situ hybridization (ISH).
(XLSX)

S4 Table Comprehensive ultrasound remarks during pregnancy. Animals C and M did not have any notable findings.
(XLSX)

S5 Table Statistics of in-utero growth trajectories of Abdominal circumference (AC), Biparietal Diameter (BPD), Femur length (FL), and Head circumference (HC) compared between Cohorts II, IV, and V. Slope estimates (95% CI) for AC, BPD, FL, and HC on log scale, stratified by cohort. 1: p-value for comparison of slopes between Cohort II vs. Cohort IV; 2: p-value for comparison of slopes between Cohort II vs. Cohort V; 3: p-value for comparison of slopes between Cohort IV vs. Cohort V.
(XLSX)
