# Supplementary figures and images for: Frequent first-trimester pregnancy loss in rhesus macaques infected with African-lineage Zika virus
Source: PLoS Pathog. 2023 Mar 28;19(3):e1011282. doi: 10.1371/journal.ppat.1011282 (PMC10081769; doi:10.1371/journal.ppat.1011282)

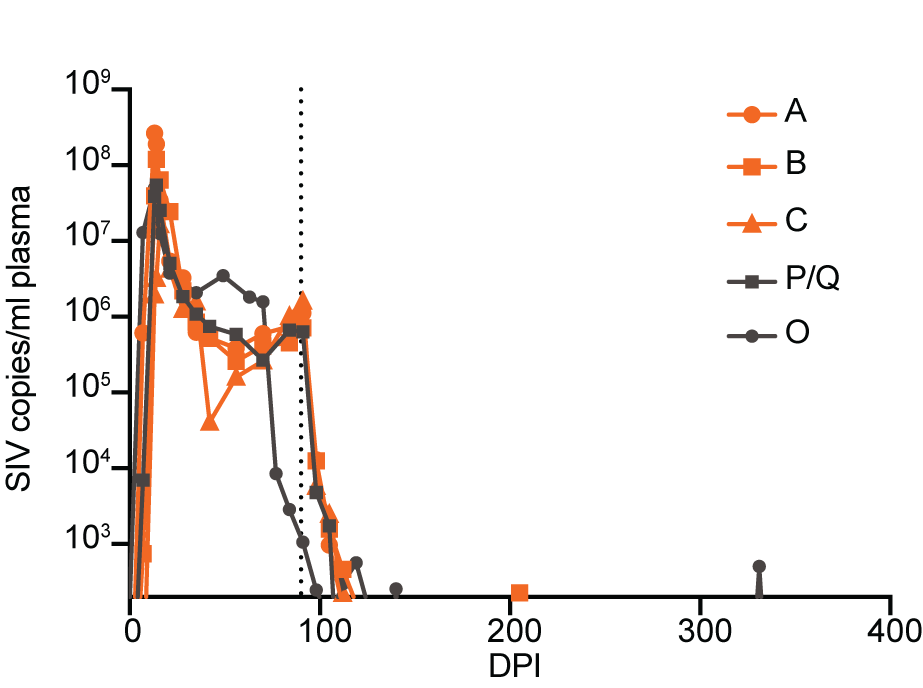

Supplement: S1 Fig — SIV viremia in macaque plasma before and after ART regimen. Copies of viral RNA were determined by SIV-specific RT-qPCR. Cohort I (SIV+/ZIKV+ +ART) pregnancies are in orange, Cohort IV (SIV+/ZIKV- +ART) pregnancies are in dark gray. The dotted line at 90 DPI indicates when animals started the 1x daily injectable combination ART regimen (TDF/FTC/DTG). Pregnancies P and Q are from the same dam and thus have the same SIV plot lines depicted here. (TIF) [file ppat.1011282.s001.tif]

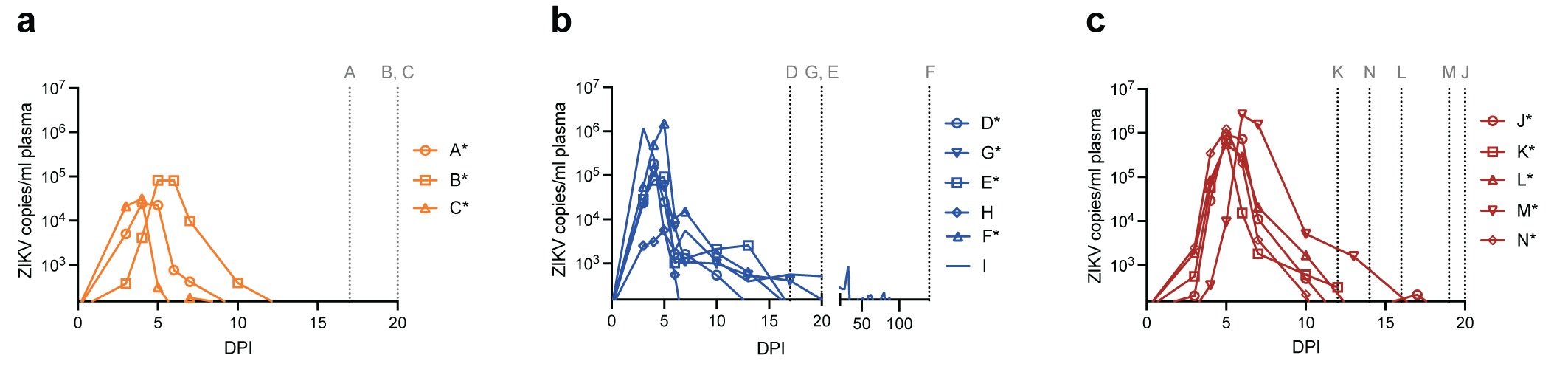

Supplement: S2 Fig — Copies of viral RNA were determined by ZIKV-specific RT-qPCR. Gray dotted lines denote the timing of pregnancy loss and the corresponding pregnancy ID(s) is/are listed above the line. Viremia corresponding to animals from (a) Cohort I are in orange, (b) Cohort II are in blue, and (c) Cohort III are in red. Plasma viremia was below the limit of quantification (LOQ) for all animals at the time of pregnancy loss except for Pregnancy K. This viral load dropped below the LOQ one day after pregnancy loss. (TIF) [file ppat.1011282.s002.tif]

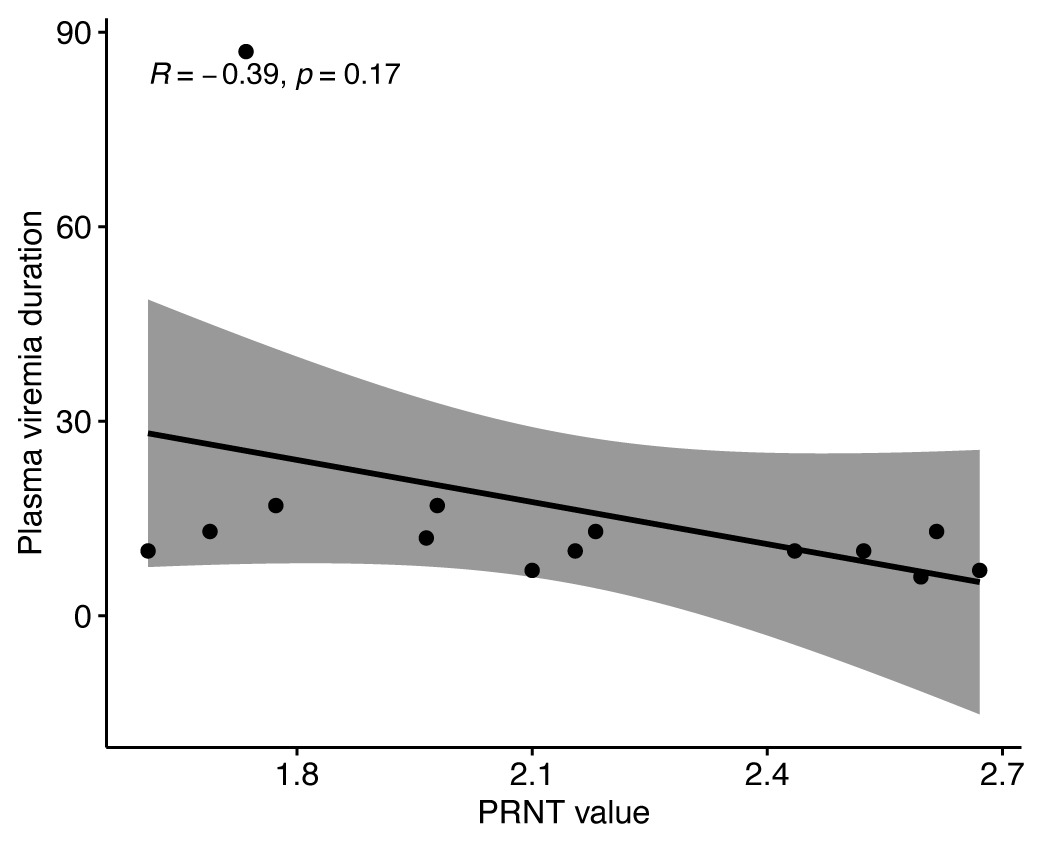

Supplement: S3 Fig — A Pearson’s correlation analysis showed no significant correlation between maternal duration of plasma viremia and PRNT90 at 17, 20, or 27 DPI (R = -0.39, p = 0.17) (TIF) [file ppat.1011282.s003.tif]

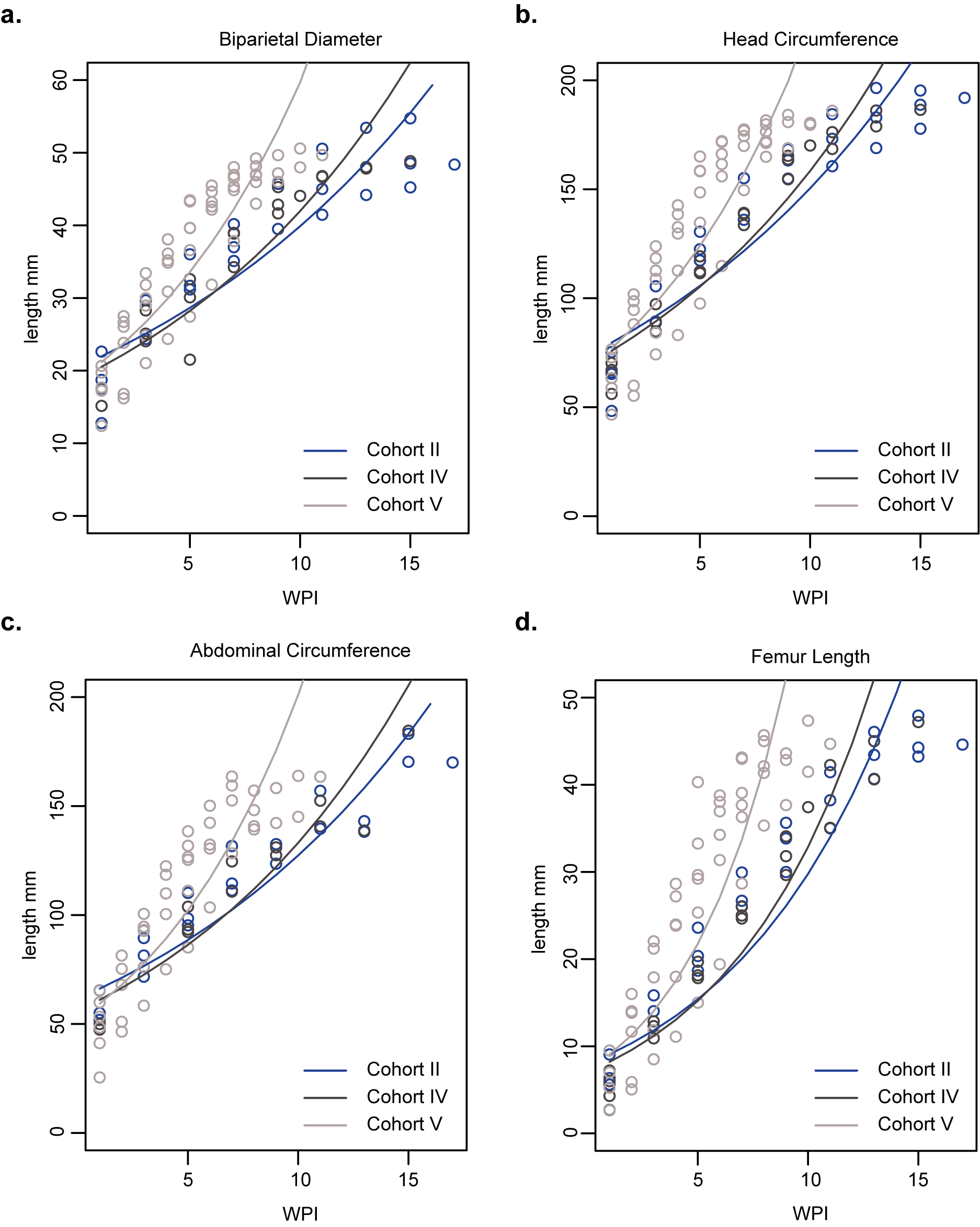

Supplement: S4 Fig — (TIF) [file ppat.1011282.s004.tif]

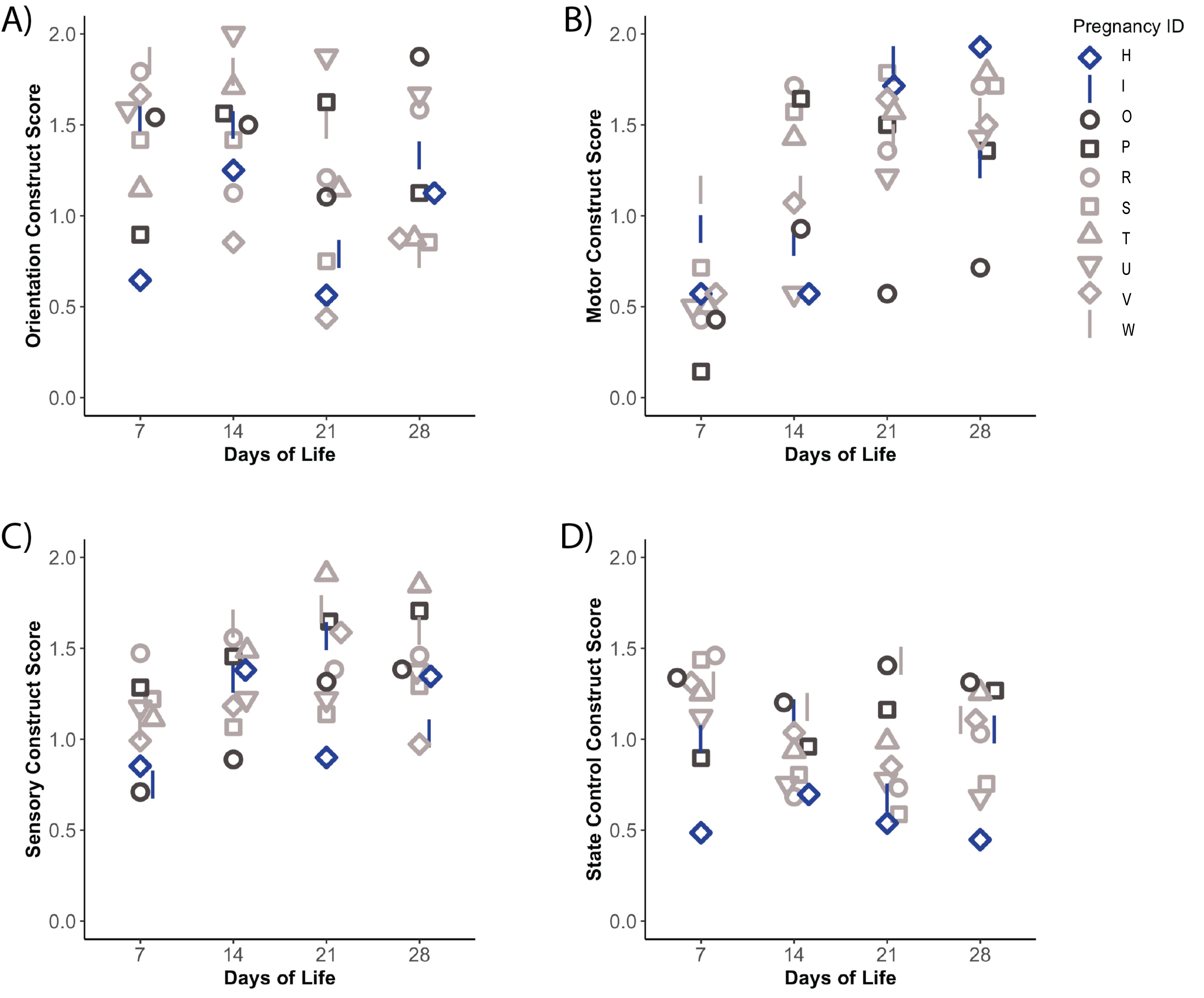

Supplement: S5 Fig — The SNAP is composed of 4 main constructs: (a) Orientation, (b) Motor maturity and activity, (c) Sensory responsiveness, and (d) State control. Cohort II (SIV-/ZIKV+ +ART) infants are in blue, Cohort IV (SIV+/ZIKV- +ART) infants are in light gray, and Cohort V (SIV-/ZIKV- +ART) infants are in dark gray. Individual infants are represented by symbols that correspond to their Pregnancy ID. (TIF) [file ppat.1011282.s005.tif]

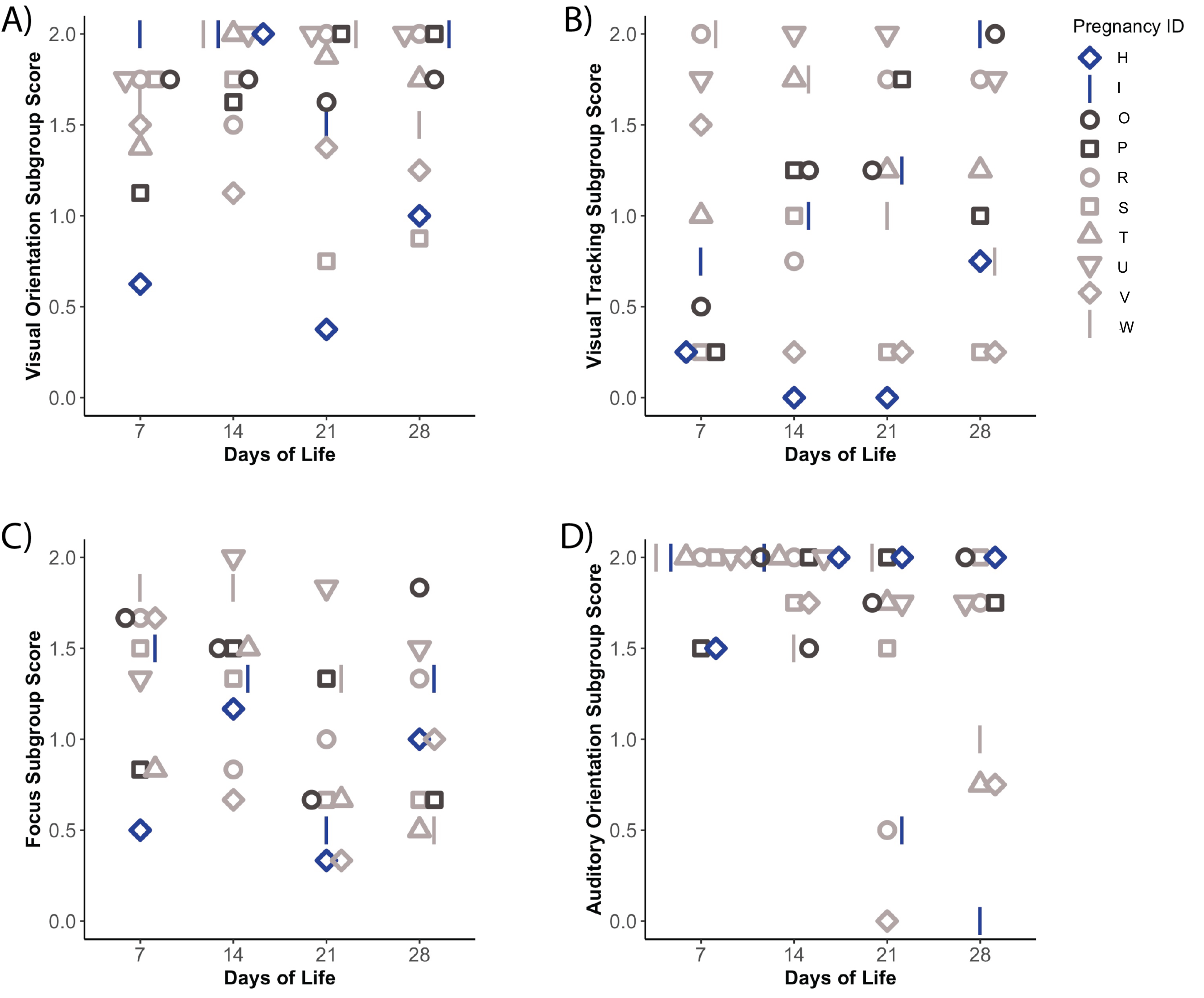

Supplement: S6 Fig — The SNAP Orientation subgroups consist of A) Visual orientation, B) Visual tracking, C) Focus, and D) Auditory orientation. Cohort II (SIV-/ZIKV+ +ART) infants are in blue, Cohort IV (SIV+/ZIKV- +ART) infants are in light gray, and Cohort V (SIV-/ZIKV- +ART) infants are in dark gray. Individual infants are represented by symbols that correspond to their Pregnancy ID. (TIF) [file ppat.1011282.s006.tif]

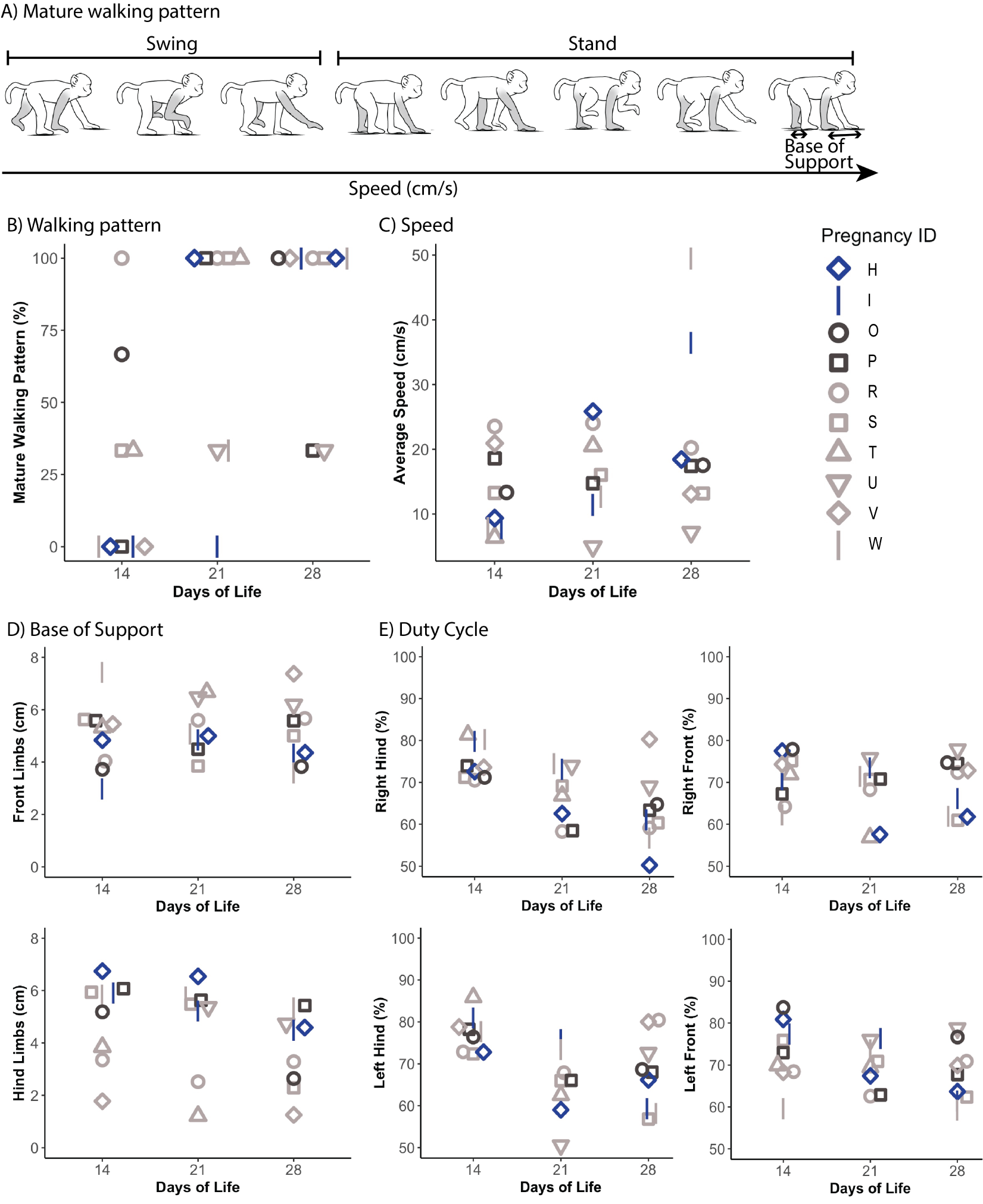

Supplement: S7 Fig — (a) Visual representation of the gait variables included mature walking pattern (where the contralateral limbs are moving through the swing and stance phase close together in timing), speed (how fast the infant walked across the catwalk), base of support (distance between right and left limbs), and duty cycle (percent of time the infant is standing on the walkway). (b) Percent of time infants use a mature walking pattern. (c) Average speed across runs, (d) Base of support for the front and hind limbs, (e) Duty cycle time the infant was standing on each limb. Cohort II (SIV-/ZIKV+ +ART) infants are in blue, Cohort IV (SIV+/ZIKV- +ART) infants are in light gray, and Cohort V (SIV-/ZIKV- +ART) infants are in dark gray. Individual infants are represented by symbols that correspond to their Pregnancy ID. (TIF) [file ppat.1011282.s007.tif]

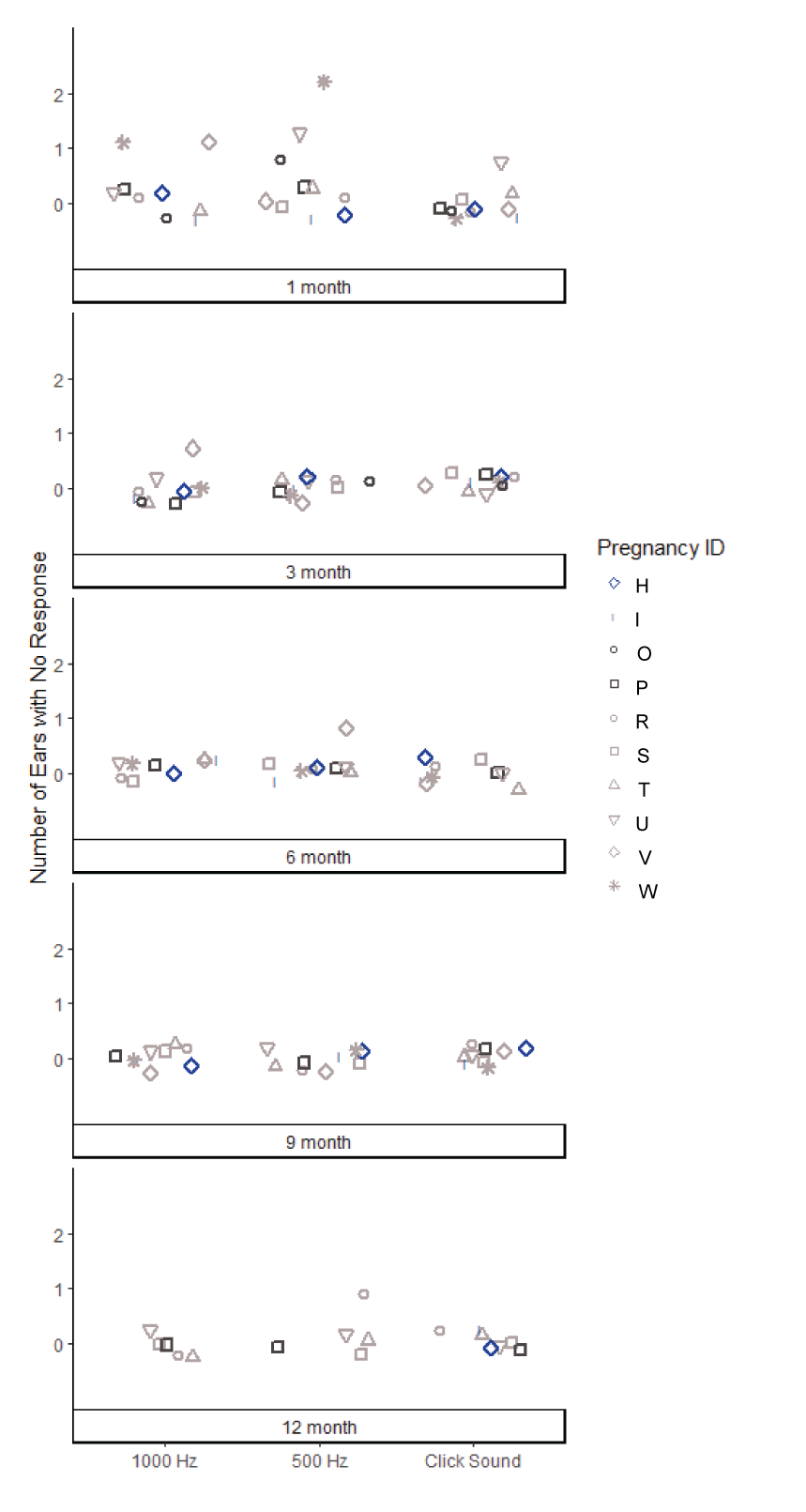

Supplement: S8 Fig — Infant macaques were tested via auditory brainstem response to click, 1000 Hz, and 500 Hz stimuli. The presence or absence of a Wave IV response for each ear was assessed and summarized for each animal at 1, 3, 6, 9, and 12 months of age. Not all the animals received testing at 12 months of age because some were not 12 months old yet. Infants with normal hearing are expected to have a score of “0” with both ears having a Wave IV auditory brainstem response at the lowest intensity level tested. Cohort II (SIV-/ZIKV+ +ART) infants are in blue, Cohort IV (SIV+/ZIKV- +ART) infants are in light gray, and Cohort V (SIV-/ZIKV- +ART) infants are in dark gray. Individual infants are represented by symbols that correspond to their Pregnancy ID. (TIF) [file ppat.1011282.s008.tif]

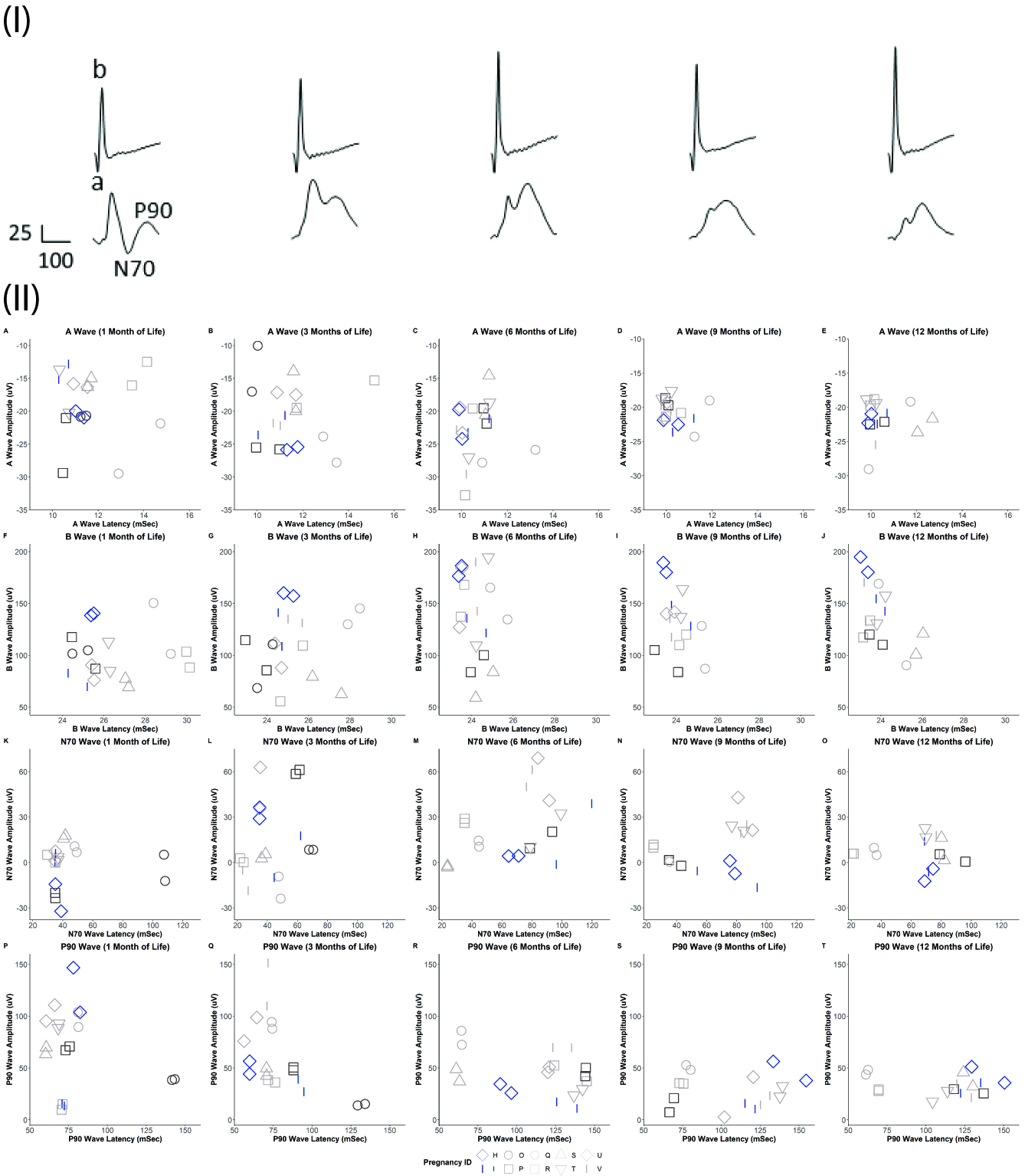

Supplement: S9 Fig — Visual function studies comprising photopic single flash electroretinograms (ERGs) and visual evoked potentials (VEPs). (I) Schematic representing the origins of the A and B waves within an ERG and the N70 and P90 waves within a VEP for each age group, aligned with the ages in part II. These representative waveforms were created from the averages of all the traces for the controls at each time point. (II) ERG A- and B-wave components were measured at 1, 3, 6, 9, and 12 months of age (A-J). The right and left eyes are plotted as individual data points. VEP N70- and P90-wave components were measured at 1, 3, 6, 9, and 12 months of age (K-T). The right and left hemispheres are plotted as individual data points. Cohort II (SIV-/ZIKV+ +ART) infants are in blue, Cohort IV (SIV+/ZIKV- +ART) infants are in light gray, and Cohort V (SIV-/ZIKV- +ART) infants are in dark gray. Individual infants are represented by symbols that correspond to their Pregnancy ID. (TIF) [file ppat.1011282.s009.tif]

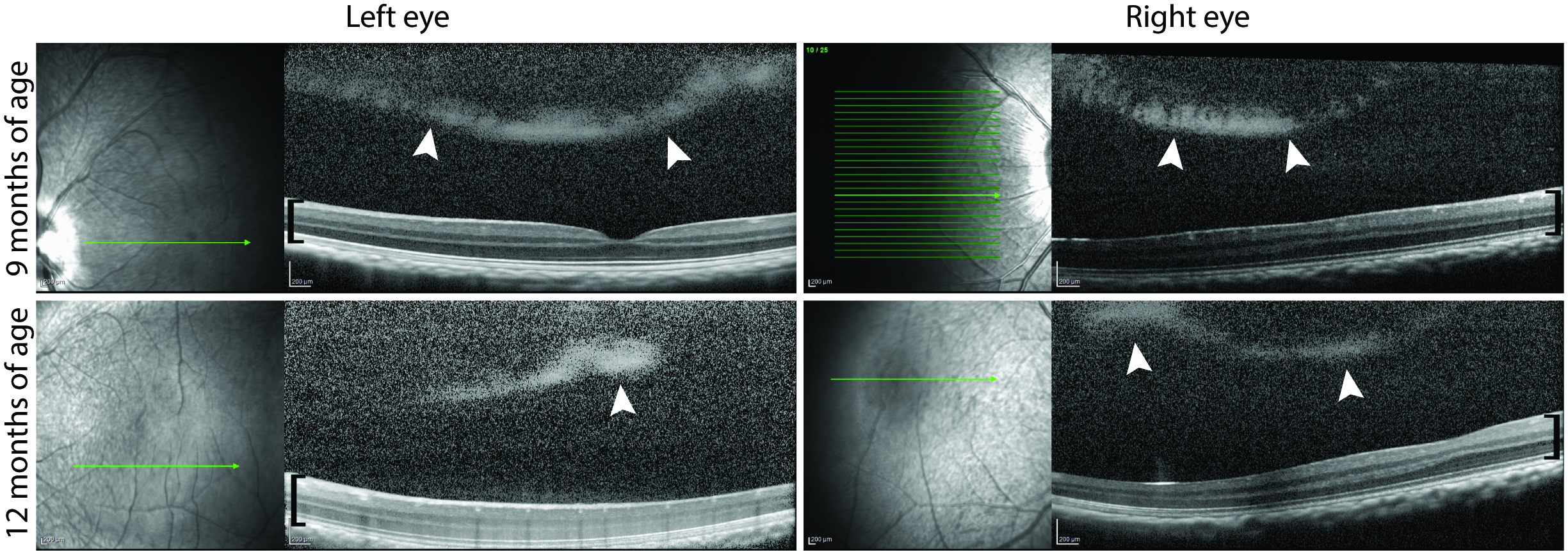

Supplement: S10 Fig — Optical coherence tomography images of the infant from Pregnancy H at 9 at 12 months of age. Vitreous opacities, or “clumping”, is seen in both right and left eyes, indicated by the white arrows. The retinal layers are denoted with a black bracket. The green arrow shows the relation of the retinal section shown in relation to the fovea. (TIF) [file ppat.1011282.s010.tif]

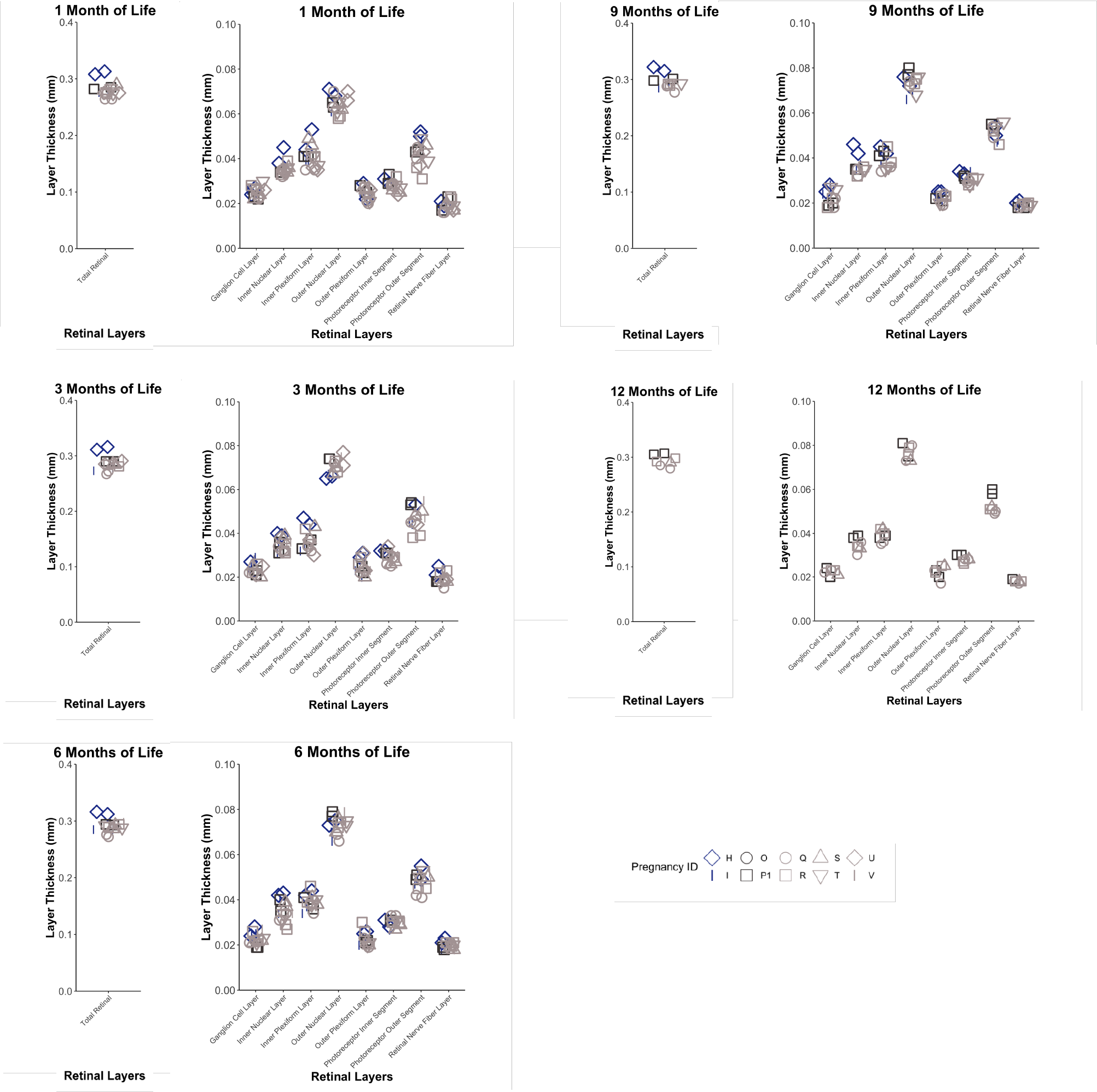

Supplement: S11 Fig — Retinal layer thicknesses were measured by optical coherence tomography (OCT) in infants at 1, 3, 6, 9, and 12 months of age. Total retinal thickness, choroidal thickness, and the thickness of individual retinal layers (ganglion cell layer, inner nuclear layer, inner plexiform layer, outer nuclear layer, outer plexiform layer, photoreceptor inner segment, photoreceptor outer segment, retinal nerve fiber layer) were determined by segmentation. Cohort II (SIV-/ZIKV+ +ART) infants are in blue, Cohort IV (SIV+/ZIKV- +ART) infants are in light gray, and Cohort V (SIV-/ZIKV- +ART) infants are in dark gray. Individual infants are represented by symbols that correspond to their Pregnancy ID. (TIFF) [file ppat.1011282.s011.tiff]
